# Biomarkers of GH deficiency identified in untreated and GH-treated Pit-1 mutant mice

**DOI:** 10.3389/fendo.2025.1539797

**Published:** 2025-04-30

**Authors:** Sarmed Al-Samerria, Huiting Xu, M. Elena Diaz-Rubio, Joseph Phelan, Chi Su, Keer Ma, Anna Newen, Kiana Li, Sayaka Yamada, Ariel L. Negron, Fredric Wondisford, Sally Radovick

**Affiliations:** ^1^ Department of Pediatrics, University of Arizona College of Medicine, Phoenix, AZ, United States; ^2^ Department of Medicine, Robert Wood Johnson Medical School, Rutgers, The State University of New Jersey, New Brunswick, NJ, United States; ^3^ Rutgers Cancer Institute, Robert Wood Johnson Medical School, Rutgers, The State University of New Jersey, New Brunswick, NJ, United States; ^4^ Department of Medicine, University of Arizona College of Medicine, Phoenix, AZ, United States

**Keywords:** growth hormone deficiency (GHD), biomarkers, PIT-1 mutation, metabolomics, GH treatment, energy metabolism, sex differences

## Abstract

**Background:**

Growth Hormone Deficiency (GHD) is marked by insufficient growth hormone (GH) production, leading to disruptions in growth and metabolism. Its diagnosis is challenging due to the lack of sensitive, specific tests. To address this, we used a novel mouse model with a POU1F1 (Pit-1) gene mutation (K216E). This study aimed to identify metabolic biomarkers of GHD and assess their responsiveness to GH therapy, alongside pathway analysis to uncover disrupted metabolic pathways.

**Methods:**

The Pit-1^^K216E^ mouse model was validated for GHD through assessments of GH production, growth, and body composition. Metabolomic profiling was conducted to identify biomarkers, while pathway analysis examined disrupted metabolic pathways and their response to GH treatment. This approach aimed to improve understanding of GHD’s metabolic impact and potential therapeutic strategies.

**Results:**

The assessment of the Pit-1^^K216E^ mouse confirmed GHD, as evidenced by reduced GH production and altered body composition. Metabolomic profiling identified three distinct biomarker groups associated with GHD: (1) GHD Biomarkers, found exclusively in GH-deficient mutant mice but absent in WT controls; (2) GH Treatment Responsive Biomarkers, which were altered in GH-deficient mutant mice (GHD) and further modulated following GH treatment, reflecting a response specific to the GHD condition and its treatment, but not observed in WT mice; and (3) GH Treatment-Specific Responsive Biomarkers, observed exclusively in the GHD condition after GH therapy. Pathway analysis revealed significant disruptions in purine metabolism, amino acid metabolism, and protein synthesis, with notable sex-specific differences. Male mice exhibited imbalances in taurine and hypotaurine metabolism, while female mice showed disruptions in tyrosine metabolism and mitochondrial function, highlighting sex-dependent metabolic responses to GHD and GH therapy.

**Conclusion:**

The Pit-1^^K216E^ mouse model offers a robust platform for exploring GHD’s molecular mechanisms. The identification of distinct, sex-specific metabolic biomarkers provides insights into GHD-related metabolic disruptions and supports personalized management strategies. These findings establish a framework for leveraging metabolic biomarkers to enhance the diagnosis and monitoring of GHD, with promising applications for future human studies and therapeutic strategies.

## Introduction

GHD is a clinical syndrome in which patients exhibit inadequate secretion of GH from the somatotrope cells in the pituitary gland. GHD can appear as an isolated growth hormone deficiency (IGHD) or in combination with other pituitary hormone deficiencies, known as CPHD. In children, it’s a common endocrine cause of growth failure and short stature (SS), with a reported incidence ranging from 1:4,000 to 1:10,000 (1–3). GHD can be exhibited at different stages of life and can lead to dramatic growth impairment and developmental delays if left untreated (4, 5).

Typically, GHD manifests after the first year of life in children, although severe cases can appear earlier during infancy (6). It is also linked to delayed bone development and metabolic disturbance (7). Given the crucial role of GH in numerous biological processes including protein synthesis and amino acid degradation, insufficient secretion of GH can result in delayed puberty, decreased muscle mass, and metabolic disorder (8). In adults, GHD can result from childhood-onset GHD persisting into adulthood or can cause acquired GHD due to factors such as pituitary tumors, traumatic brain injury, or radiation therapy (9, 10).

The diagnosis of GHD is associated with multiple challenges due to the complex nature of the disease, the limitations of current diagnostic methods, and the lack of a standard test that can be used as a reliable diagnostic method. According to the GH Research Society (GHRS), The current diagnostic approach involves the evaluation of auxologic parameters such as height, and weight, followed by a biochemical assessment of serum levels of GH and IGF-I and comprehensive radiological evaluation (4, 11, 12). However, clinical evaluation and interpretation of GH and IGF-I levels lack sensitivity and specificity, leading to diagnostic ambiguity (3, 13). Furthermore, provocative tests, which stimulate GH secretion to assess pituitary function, are commonly used to diagnose GHD. However, these tests have several limitations. Firstly, no universally agreed-upon cutoff level discriminates a normal response from a deficient response to provocative stimuli. This lack of standardized criteria can result in variability in test interpretation and diagnostic accuracy. Furthermore, GH-stimulating tests have low specificity, resulting in false-positive results and unnecessary treatment in some cases (2, 3). Sexual dimorphism in GH production plays a key role in metabolic regulation, body composition, and growth (14). The analysis of GH production revealed a clear sexual dimorphic pattern in both rodents and humans. In rodents, males appear to have more pulsatile GH secretion, in contrast, females have a more continuous pattern (15). This sex-specific secretion pattern is also seen in humans, though it is less pronounced (14). Understanding these differences is critical when assessing the efficacy of GH treatment, as responses to therapy can vary between sexes due to this underlying dimorphism. Therefore, identifying sex-specific biomarkers is necessary for monitoring the response to GH treatment and designing effective treatment strategies. Metabolomic profiling has become necessary in the quest for the identification of potential biomarkers (metabolites), in the context of metabolic and endocrine disorders such as GHD (16–19). Several studies have been conducted employing various experimental approaches, involving both human and animal models. These investigations aim to unveil distinctive metabolic signatures associated with GHD, offering insights into its pathophysiology, and facilitating accurate diagnosis. However, despite the extensive research, human studies are associated with significant challenges, including limitations in sample collection, variability in patient characteristics, and ethical considerations for GH therapy (18, 20–23).

Previously, our laboratory identified a patient with SS who was found to have a point mutation in one allele of the POU1F1 gene (POU domain, class 1, transcription factor 1), also known as Pit-1. This mutation led to substituting lysine (K) with glutamic acid (E) at position 216 in the Pit-1 protein, encoded by the POU1F1 gene. Despite having measurable basal GH levels, proactive test evaluations revealed that the patient’s GH response to insulin-induced hypoglycemia and glucagon stimulation was deficient, with a peak GH release that was notably low. The patient also showed partial prolactin (PRL) deficiency, with normal basal PRL levels but no significant increase following thyrotropin-releasing hormone (TRH) stimulation. Initially, thyroid function appeared normal, but secondary hypothyroidism developed later, as evidenced by a low serum T4 level and a diminished TSH response to TRH stimulation (24).

Using advanced gene-editing techniques, we employed CRISPR-Cas9 technology to generate a novel mouse model, referred to as the Pit-1^^K216E^ mutant mouse. This mouse model carries the specific point mutation identified in the *PIT-1* gene, closely mirroring the genetic anomaly observed in the CPHD patient. We proposed that this model will provide an invaluable tool for identifying potential biomarkers for the CPHD condition, facilitating the advancement of diagnostic methods and treatment strategies for GH replacement therapy. Furthermore, employing metabolomic approaches allows us to investigate the metabolic profile of GHD, enabling the characterization of metabolic changes associated with the condition and the evaluation of responses to GH treatment.

Our first aim is to characterize the metabolic profile of GHD in our mouse model and to study the effects of GH therapy on *Pit-1* mutation. We hypothesize that certain metabolic pathways will exhibit dysregulation in our mouse model compared to the wild-type, offering diagnostic specificity and sensitivity.

Building upon the metabolic characterization, our second aim is to identify potential biomarkers that can serve as diagnostic indicators for GHD. Lastly, we endeavor to assess the efficacy of these identified biomarkers in monitoring treatment response to GH therapy. This involves comparing the dynamics of metabolite changes in response to GH treatment in our mouse models, both GH-treated and untreated, alongside wild-type counterparts.

The identified metabolic biomarkers not only provide insights into the metabolic consequences of GHD but also have potential clinical applications in humans. These biomarkers could aid in the early diagnosis of GHD, improve disease monitoring, and help assess treatment responses, addressing current challenges in managing GHD patients.

## Materials and methods

**Figure 1:**
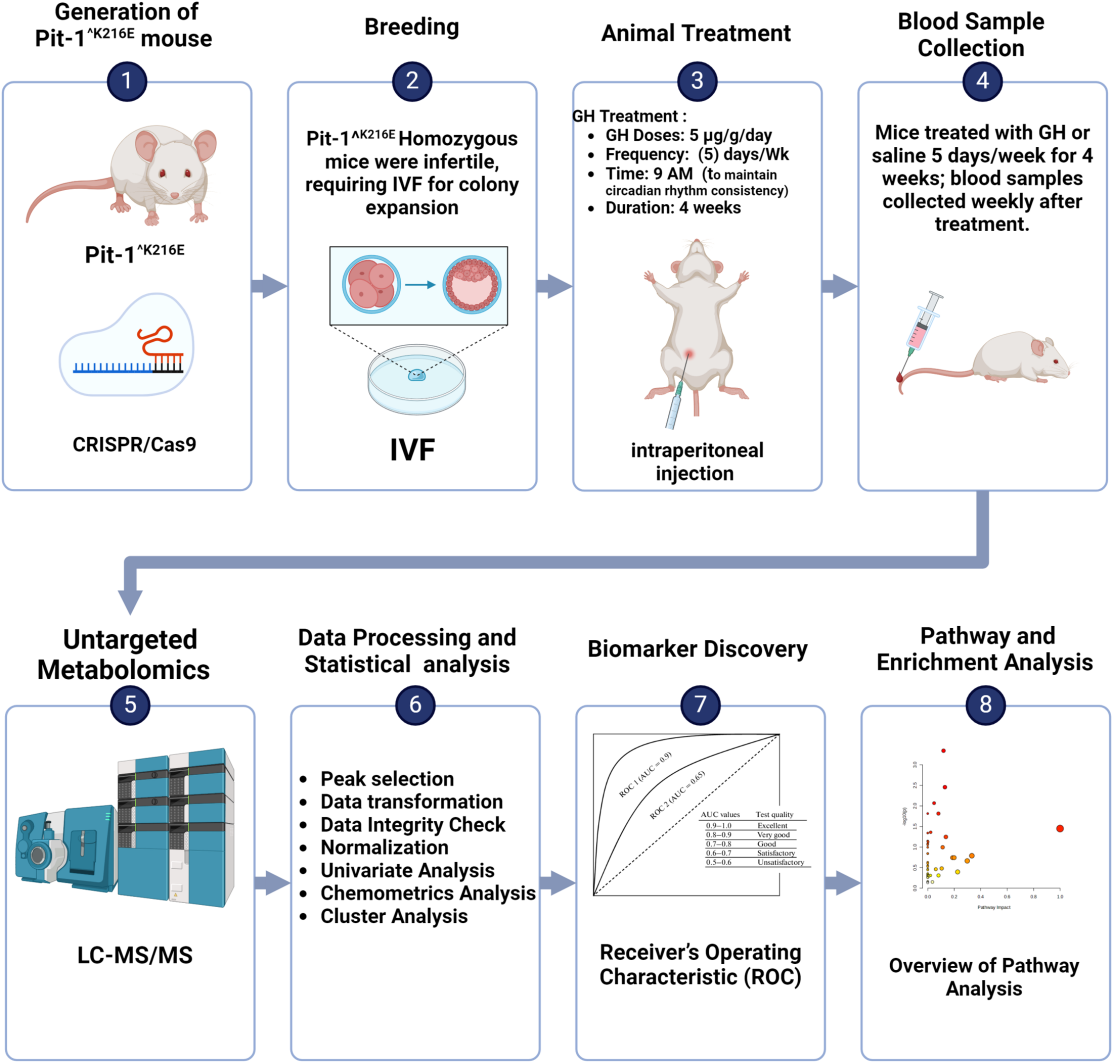
Schematic depiction of the comprehensive workflow used in metabolomics analysis to identify potential biomarkers associated with GHD. This figure was created using BioRender.com.

The workflow chart summarizing our experimental approach to investigate the metabolomic profiles under GHD conditions using Pit-1^^K216E^ mouse models, which will be referred to as mutant (Mut) throughout this study, is illustrated in **Figure 1**. This figure provides an overview of the key steps, including the creation of mutant mice, breeding, GH treatment, blood sample collection, untargeted metabolomic analysis, data processing, and the subsequent discovery of biomarkers.

### Generation of the Pit-1^^K216E^ mutant mouse model using CRISPR-Cas9

The Mut mice were generated using the CRISPR-Cas9 technique. C57BL6/J embryos were subjected to microinjection with a mixture comprising Cas9 protein (IDT Coralville, Iowa, USA), a single-guide RNA (sgRNA) (MilliporeSigma, t. Louis and Burlington, MA), and a single-stranded oligodeoxynucleotide (ssODN) (IDT Coralville, Iowa, USA) containing homology arms and carrying the K216E mutation. The sgRNA sequence employed was AGAAGGTGGGAGCAAACGAAAGG (with the PAM site underlined), and the donor oligo sequence was as follows:

TAAATACGGACTCCGTGTGAACATGATGTTGTTCTTTCTCTAGTAAGTTAAGGATCGCAAAGGAATACCTGATGGTTGTCCTCCG**c**T**c**CCTCTTTCTTTCGTTTGCTCCCACCTTCTCATTGTACAAAGCTGGAATGTAGAAAGGGGAGAATAAGAACTAGGAATTTTAAACTATCATTCTTTT (with the K216E mutation sequence in lower case bold and the PAM site change in upper case bold). Founders were initially screened using PCR and digestion with the AciI restriction enzyme (NEB, Ipswich, MA, USA, Cat.No. R0551S), followed by confirmation through Sanger sequencing.

Confirmed founders were then bred, and their offspring were screened using PCR primers (Pit-1 Sense 5’-ACCTACTTGGCAAACATTTGAGAC-3’ and Pit-1 antisense 5’-ATTCACCTCATAATAATGTTGCTCTTATAC-3’). The presence of the AciI restriction site or the results of Sanger sequencing of PCR products were used for verification.

### Animal care and assessment of auxological parameters

The animals in this study were housed in the controlled environment of the Child Health Institute of New Jersey (CHI-NJ) vivarium. To facilitate their adaptation to the experimental conditions, a mandatory 72-hour acclimation period was provided. Environmental conditions were strictly monitored, with the vivarium maintained at a temperature range of 65-75 (18-23 °C) and a humidity level between 40-60%. A 12-hour light/dark cycle (lights on at 6:00 a.m.) was implemented to regulate circadian rhythms. Mice had ad libitum access to a standard rodent diet (RD: 5058 PicoLab^®^ Rodent Diet 20) and unrestricted access to water. Housing conditions consisted of groups of five mice per cage, provided with environmental enrichment to encourage physical and cognitive stimulation, all housed in well-ventilated racks.

The homozygous Mut mice, distinguished by their fragility, were provided with specialized care throughout the study, with a key focus on maintaining their proximity to their mother for additional support during early development. In addition, these mice were found to be infertile, likely due to abnormal growth affecting their sexual function rather than impairments in sex hormones like FSH and LH. Colony propagation was achieved through *in vitro* fertilization (IVF) to ensure a stable and consistent population of homozygous mutants for the study.

Mice subjected to GH treatment in this study received specialized care to minimize stress and optimize acclimation to the study conditions.

Two weeks before commencing the GH treatment, the mice underwent a careful and systematic acclimation process. During this acclimation period, the mice were regularly handled to ensure they became accustomed to the presence of the mouse handlers and the handling techniques. To ensure the mice’s comfort and minimize stress during the non-anesthetic body length measurements, a gentle and careful approach was employed. The procedure involved cradling the mouse with one hand behind its neck and the other hand gently holding its tail straight. Subsequently, a Vernier caliper micrometer was used to measure the mouse’s length. The caliper’s jaws were opened to their maximum extent, positioned at the mouse’s nose, and then gently closed until they touched the anus. The precise length measurement was then recorded to the nearest 0.1 cm. All procedures were performed according to the Rutgers University protocol approved by the Institutional Animal Care and Use Committee at the Child Health Institute, Rutgers University, New Brunswick, NJ.

### Pituitary gland gene expression profile in the Pit-1^^K216E^ mice

Total mRNA was extracted from the pituitary gland harvested at 12-15 weeks of age, using Trizol (Invitrogen, Carlsbad, CA). Briefly, 2 μg of RNA were reverse transcribed (iScript cDNA Synthesis kit; Bio-Rad) to produce cDNA. cDNA obtained from 50 ng of total RNA was used in each reaction; 25-μL PCRs were performed using the IQ SybrGreen supermix (Bio-Rad). Reactions were measured using the MyiQ qRT-PCR machine (Bio-Rad). Primer set for *Gh* (sense 5′-TCCTCAGCAGGATTTTCACC-3′ and antisense 5′- CATGTTGGCGTCAAACTTGT-3′), and *Prl* (sense 5’- AGCCCCCGAATACATCCTAT3’ and antisense 5’- ATCCCATTTCCTTTGGCTTC-3’), and *Tshβ* (sense 5’- GTGCTGGGTATTGTATGACACG-3’ and antisense 5’-CTGGTATTTCCACCGTTCTGTAG-3’). *Actb* was used as a housekeeping gene with the following primers: sense (5′-CCAGTTGGTAACAATGCCATG-3′) and antisense (5′-GGCTGTATTCCCCTCCATCG-3′).

### Hormones level assessment

For mouse model characterization, blood samples were collected at 12–15 weeks of age, and serum levels of GH, PRL, and TSH were measured using the MILLIPLEX^®^ Mouse Pituitary Magnetic Bead Panel (Millipore Sigma, Burlington, MA, USA) according to the manufacturer’s instructions. This assay offers high sensitivity (≤1 pg/mL for GH and PRL and ≤4 pg/mL for TSH) and specificity due to its advanced multiplexing technology, which minimizes cross-reactivity between analytes. All analyses were conducted at the NJMS Flow Cytometry and Immunology Core Laboratory. Serum T3 and T4 levels were quantified using the Mouse Tri-iodothyronine (T3) and Thyroxine (T4) ELISA Kits (Aviva Systems Biology, San Diego, CA, USA). The sensitivity for these assays is 0.12 ng/mL for T3 and 0.1 µg/dL for T4, with high specificity for their respective hormones. Manufacturer guidelines were strictly followed to ensure accuracy. Serum IGF-I levels were detected using the Mouse IGF-I ELISA Kit (Millipore/Sigma-Aldrich, RAB-0229) following the manufacturer’s instructions. This assay provides a sensitivity of 8 pg/mL and high specificity due to its monoclonal antibody-based detection. For the animal cohort involved in the GH treatment study, blood was obtained from 8-week-old experimental groups (at the end of GH treatment) via terminal cardiac puncture.

### Anatomical and histological assessment of the pituitary gland

Pituitary glands were collected, at 12-15 weeks of age, and dissected from Mut mice (aged and sex-matched). The collected pituitary glands were fixed in 4% paraformaldehyde (PFA) for a minimum of 24 hours at 4°C before being subjected to further analysis. The fixed tissues were submitted to Rutgers University’s Pathology Core for histological examination. Cross-sectional sections of pituitary glands were cut at 4µm thickness and subjected to hematoxylin and eosin (H&E) staining.

### GH replacement therapy for wild-type and mutant Pit-1^^K216E^ mice

Both wild-type (WT) and Mut mice were divided into two experimental groups to explore the effect of GH treatment. Mice received 100µl of intraperitoneal injection (ip) either of GH treatment (Prospect, East Brunswick, NJ, USA, Cat. No. CYT-202) or saline (MilliporeSigma, t. Louis and Burlington, MA) as a control. The GH dose (5 µg/g/day) was selected based on previous studies demonstrating its efficacy in restoring physiological GH levels in GH-deficient mice (23). The treatment started at 4 weeks of age and continued until 8 weeks of age, for a total treatment period of 4 weeks. Mice were treated 5 days a week, with injections administered at 9 AM. Blood samples (50µl) were collected from the tail vein in the afternoon of the same day as the last injection. Body weight and length were measured, and the mice were left to rest for 2 days before starting a new treatment cycle. Due to the fragile condition of the Mut mice, fasting was avoided before injections or sample collections to reduce stress and prevent potential health risks.

### Auxological assessment of wild-type and mutant Pit-1^^K216E^ mice

Total body length (BL) (naso-anal) was measured using a Scienceware^®^ vernier caliper (MilliporeSigma, t. Louis and Burlington, MA), and body weight (BW) was recorded weekly using a single electronic scale. Measurements for both WT and Mut mice were recorded weekly, started at 4 weeks of age, and continued until 8 weeks of age. For mice treated with GH, total body weight and length were assessed weekly starting at 4 weeks of age and continued until 8 weeks of age, marking the end of the GH treatment period.

### Effect of GH treatment on wild-type and mutant Pit-1^^K216E^ mice on body composition and metabolic rate

After completing the treatment cycle at 8 weeks of age, the experimental mice underwent body composition analysis using EchoMRI™500 Body Composition Analyzer. This method was used to assess fat and lean mass and to calculate the ratios of (fat mass to body mass) and (lean mass to body mass). To assess metabolic rate in Mut and WT mice following GH treatment, we subjected mice to a Comprehensive Lab Animal Monitoring System (CLAMS) indirect calorimetric study. Mice were individually housed with ad libitum access to food and water. A mandatory acclimation period of 24-48 hours was observed before data collection for analysis. Oxygen consumption (VO_2_) and carbon dioxide production (VCO_2_) were monitored at 15-minute intervals to evaluate the effect of GH treatment on the metabolic rate and calculate the respiratory exchange ratio. For mice subjected to metabolic parameters examination using (CLAMS), GH was administered three times per week, with injections given every second day, rather than five times weekly, to maintain GH levels while minimizing interruptions to the recording system.

### Non-targeted metabolomic analysis

40 µL methanol (ice-cold) was added to 10 µL of each serum sample. The mixture was transferred into a clean Eppendorf tube and vortexed for 30 seconds. The samples were placed at -20°C for 20min for protein precipitation, and then the samples were removed from -20°C and let sit at room temperature for 5 minutes. The samples were centrifuged for 10 minutes at 13,000 g at 4°C. The supernatant was transferred to a clean tube and a second extraction was made on the pellet by adding 200 µL 40:40:20 (v/v) methanol:acetonitrile: H2O. After 10 minutes of centrifugation at 13,000 g and 4°C, both supernatants were combined and cleaned up by using Phree Phospholipid Removal SPE cartridges (Phenomenex, Torrance, CA) according to the manufacturer’s instructions.

### LC-MS analyses

The LC-MS was performed on a Q Exactive PLUS hybrid quadrupole-orbitrap mass spectrometer coupled to a Vanquish Horizon UHPLC system (Thermo Fisher Scientific, Waltham, MA) with an XBridge BEH Amide column (150 mm × 2.1 mm, 2.5 μm particle size, Waters, Milford, MA). The HILIC separation used a gradient of solvent A (95%:5% H2O: acetonitrile with 20 mM acetic acid, 40 mM ammonium hydroxide, pH 9.4) and solvent B (20%:80% H2O: acetonitrile with 20 mM acetic acid, 40 mM ammonium hydroxide, pH 9.4). The gradient was 0 min, 100% B; 3 min, 100% B; 3.2 min, 90% B; 6.2 min, 90% B; 6.5 min, 80% B; 10.5 min, 80% B; 10.7 min, 70% B; 13.5 min, 70% B; 13.7 min, 45% B; 16 min, 45% B; 16.5 min, 100% B; and 22 min, 100% B (25). The flow rate was 300 μL/min. The column temperature was set to 25°C. The autosampler temperature was set to 4°C, and the injection volume was 5 μL. MS scans were obtained in negative and positive ionization mode with a resolution of 70,000 at m/z 200, in addition to an automatic gain control target of 3 x 106 and m/z scan range of 72 to 1000. Metabolite data was obtained using the MAVEN software package mass accuracy window: 5 ppm) (26). The metabolites detected in positive ion mode were pooled with those metabolites detected in negative ion mode (post-identification) to create a comprehensive metabolites profile for each sample.

### Statistical analysis

Statistical analyses of the Mut mouse model characterization and the effects of GH treatment in both WT and Mut mice were conducted using Prism GraphPad 10 software (GraphPad Software, Inc., San Diego, CA). All metabolomic analyses were performed using MetaboAnalyst 6.0 (Xia Lab, McGill University, Montreal, QC, Canada). Metabolomic data were processed using a standardized normalization approach applied consistently across all samples, regardless of sex. Normalization included sample normalization by median, log10 transformation (base 10), and Pareto scaling. Principal Component Analysis (PCA) was employed to visualize group separations, with the robustness of PCA models validated using cross-validation and permutation tests to prevent overfitting. Receiver Operating Characteristic (ROC) analysis, using a non-parametric method, evaluated the diagnostic potential of biomarkers, with criteria including an area under the curve (AUC) ≥ 0.8 and determination of optimal cut-off points with thresholds set at a log2 fold change ≥ 1.5, p-value ≤ 0.05. Additionally, Cohen’s d was calculated to assess the magnitude of metabolic differences, using the standard deviation (SD) of each metabolite.

Pathway analysis was conducted using the Kyoto Encyclopedia of Genes and Genomes (KEGG) database to identify metabolic pathways disrupted in GHD and their response to GH therapy. Pathways were prioritized based on enrichment analysis and impact scores, providing insights into the biological relevance of identified metabolites.

## Results

### Characterization of the Pit-1^^K216E^ mouse model

**Figure 2:**
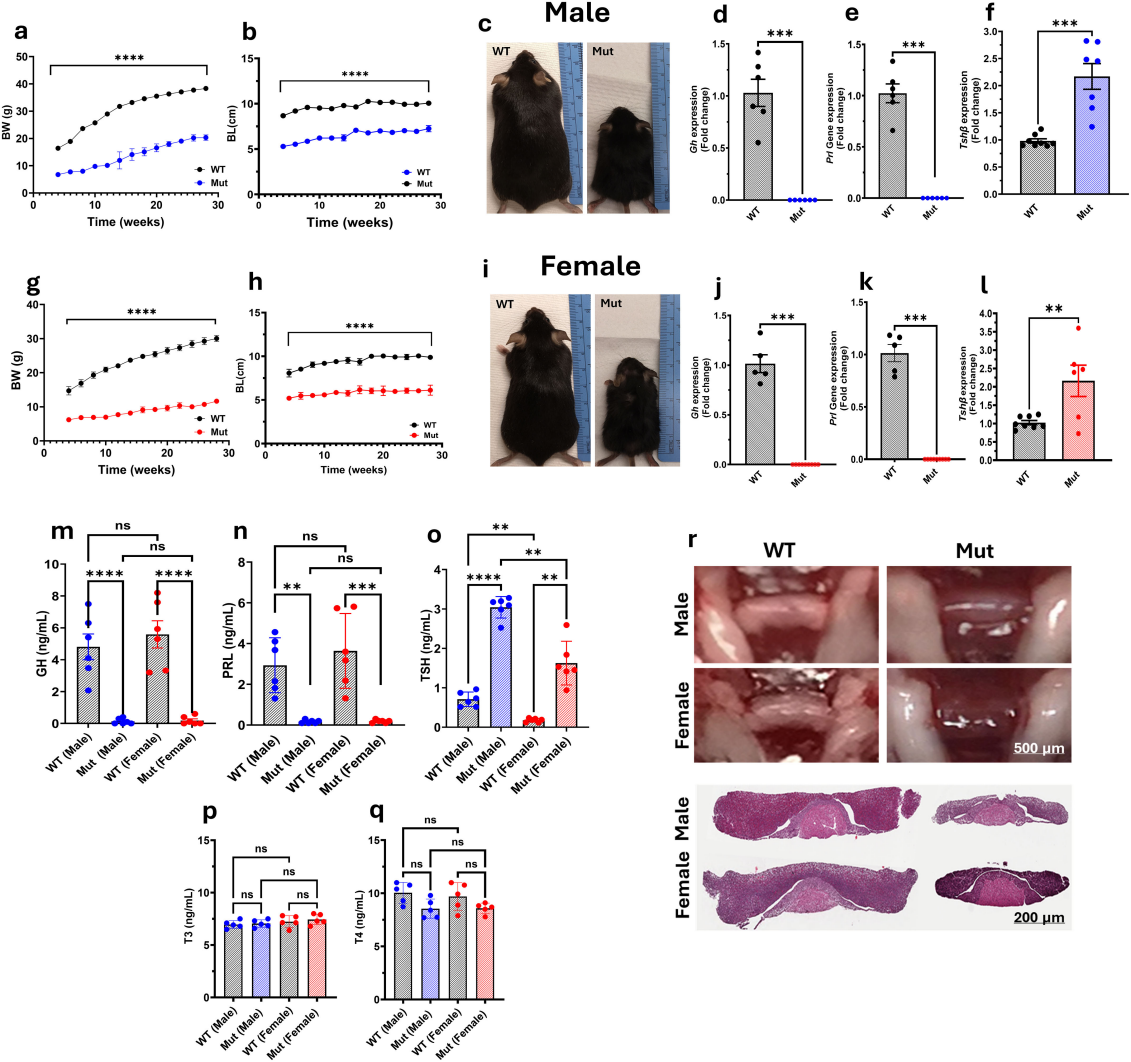
Characterization of the Pit-1^^K216E^ Mutant Mouse Model. BW and BL were measured from 4 to 28 weeks in males **(a, b)** and females **(g, h)**. Representative images at 8 weeks show body size differences in males **(c)** and females **(i)**. Gene expression of Gh, Prl, and Tshβ in WT and Mut males **(d-f)** and females **(j-l)**. Serum hormone levels: GH **(m)**, PRL **(n)**, TSH **(o)**, T3 **(p)**, and T4 **(q)**. Pituitary morphology **(r)**: gross anatomy (upper) and H&E staining (lower), showing hypoplasia in Mut mice. Scale bars: 500 μm (gross), 200 μm (H&E). Data: Mean ± SEM (n = 5-13). Statistical significance was determined using an unpaired t-test followed by the Mann-Whitney test. Significance levels are indicated as follows: ns (not significant), **p < 0.01, ***p < 0.001, ****p < 0.0001.

In this study, WT and Mut mice were monitored from 4 to 28 weeks of age, as shown in **Figure 2**. In males, Mut mice exhibited a significant reduction in BW and BL compared to WT controls, with statistical significance for both parameters observed from 4 weeks of age onward (p < 0.0001) (**Figures 2a, b**). At 4 weeks, WT males had an average BW of 16.84 ± 0.61 g and a BL of 8.67 ± 0.49 cm, whereas Mut mice had an average BW of 6.75 ± 0.44 g and a BL of 5.28 ± 0.31 cm. By the end of the study (28 weeks), these differences remained significant, with WT males reaching an average BW of 38.34 ± 1.12 g and a BL of 10.06 ± 0.23 cm, compared to Mut mice, which had an average BW of 20.1 ± 1.4 g and a BL of 7.25 ± 0.681 cm.

Similarly, female Mut mice showed significantly lower BW and BL compared to their WT counterparts (p < 0.0001) for both BW and BL (**Figures 2g, h**), respectively. At 4 weeks of age, WT mice had an average BW of 18.75 ± 0.27 g and a BL of 8.07 ± 0.93 cm, whereas Mut mice had an average BW of 6.23 ± 0.26 g and a BL of 5.19 ± 0.06 cm. The reduced growth pattern in female Mut mice persisted until 28 weeks of age, with WT mice reaching an average BW of 30.05 ± 1.03 g and a BL of 9.88 ± 0.15 cm, while Mut mice had an average BW of 11.7 ± 0.68 g and a BL of 6.12 ± 0.26 cm. Furthermore, representative images of male (**Figure 2c**) and female (**Figure 2i**) WT and Mut mice at 8 weeks of age, corresponding to the onset of puberty, further illustrate these differences.

Male and female Mut mice did not exhibit any sexual activity throughout the study, despite multiple attempts to pair them at different stages of their life. This lack of sexual activity is believed to be due to impairments in their growth trajectory rather than deficiencies in reproductive hormones like FSH and LH.

To validate further our mouse model, qPCR analysis was performed to examine the expression levels of *Gh*, *Prl*, and *Tshβ* genes in the pituitary glands of male (**Figures 2d-f**) and female (**Figures 2j-l**) mice. In male Mut mice, *Gh* (**Figure 2d**) and *Prl* (**Figure 2e**) expression levels were significantly lower compared to WT controls (p < 0.0025 and p < 0.0008), respectively. In contrast, *Tshβ* (**Figure 2f**) expression showed significant upregulation (p < 0.0003). A similar pattern was observed in female Mut mice, where the expression of *Gh* (**Figure 2j**) and *Prl* (**Figure 2k**) was significantly lower (p < 0.0003 for both) compared to WT controls, and *Tshβ* (**Figure 2l**) expression remained upregulated (p < 0.0096).

Serum hormone levels were measured to evaluate the systemic impact of the mutation. GH levels (**Figure 2m**) were significantly lower in both male and female Mut mice compared to the WT control (p < 0.0001 for both). Specifically, GH serum levels in WT males and females were 4.8 ± 1.97 ng/mL and 5.59 ± 2.09 ng/mL, respectively, whereas Mut males and females had significantly lower levels of 0.133 ± 0.175 ng/mL and 0.183 ± 0.256 ng/mL, respectively. PRL levels (**Figure 2n**) also showed a significant decrease (p < 0.0001) in both male and female Mut mice, with levels of 0.167 ± 0.082 ng/mL and 0.183 ± 0.075 ng/mL, respectively, compared to 2.93 ± 1.35 ng/mL in WT males and 3.64 ± 1.8 ng/mL in WT females. Interestingly, serum TSH levels (**Figure 2o**) were significantly elevated in male Mut mice (3.043 ± 0.273 ng/mL) compared to WT males (0.709 ± 0.075 ng/mL, p < 0.0001), and in female Mut mice (1.62 ± 0.554 ng/mL) compared to WT females (0.178 ± 0.02 ng/mL, p < 0.0066). To evaluate the effect of TSH elevation, triiodothyronine (T3) (**Figure 2p**) and thyroxine (T4) (**Figure 2q**) levels were measured in the serum. No significant differences were observed between WT and Mut mice in either sex.

Lastly, histological analysis of the pituitary glands (**Figure 2r**) revealed morphological differences between WT and Mut mice in both males and females, providing further evidence of the impact of the mutation on pituitary structure.

### Assessment of growth, development, and body composition in Pit-1^^K216E^ mutant mice in response to GH treatment

To investigate the impact of GH treatment on the growth of Mut mice, measurements were recorded starting at 4 weeks of age and continued until 8 weeks of age, corresponding with the duration of GH treatment. A control group treated with saline was included for comparison purposes and will be referred to as the control.

**Figure 3:**
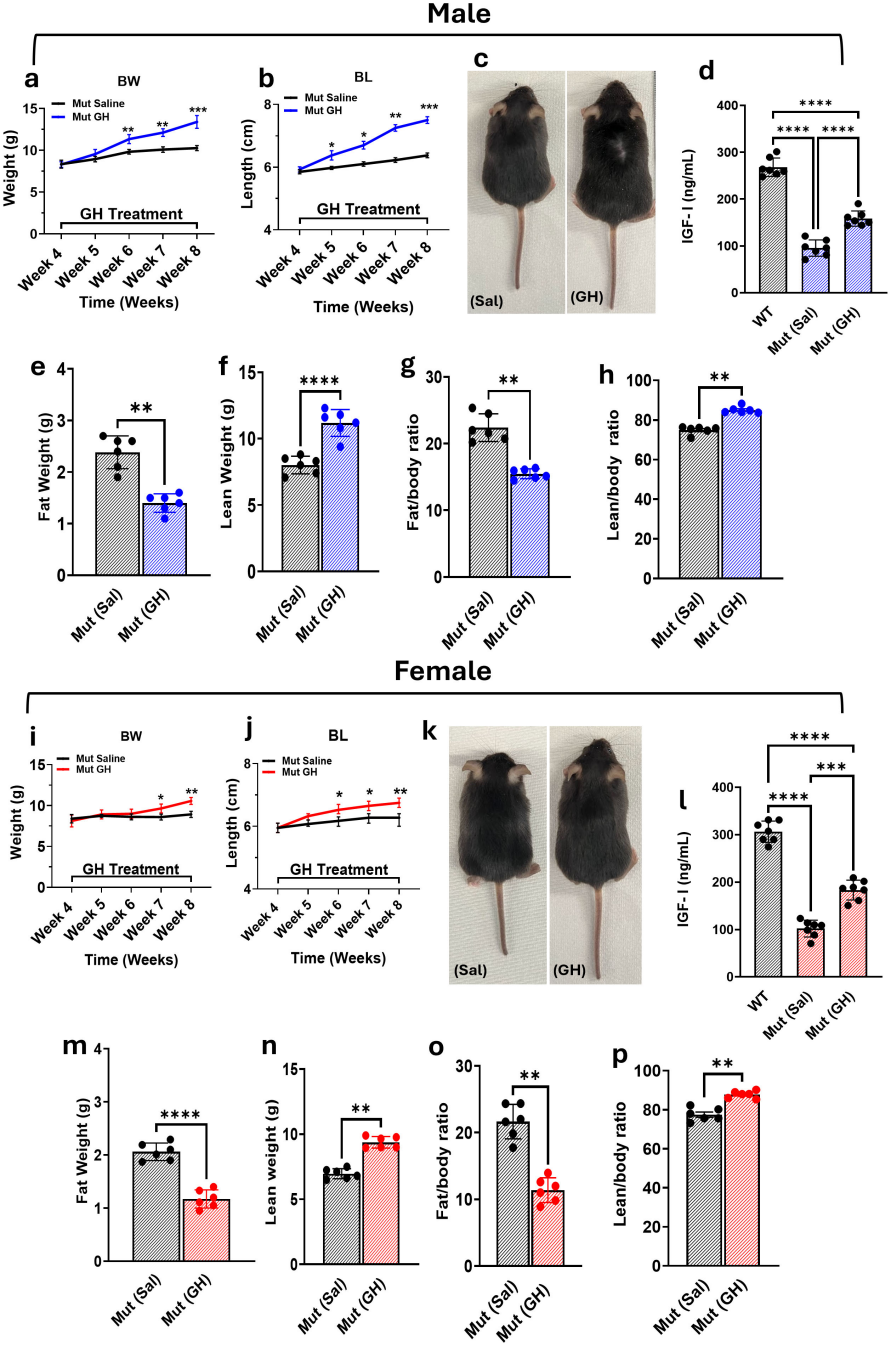
Effects of GH Treatment on Pit-1^^K216E^ Mutant Mice BW **(a, i)** and BL **(b, j)** growth curves in male and female Mut mice treated with Sal or GH for 4 weeks. Representative images **(c, k)** show size differences post-treatment. Serum IGF-1 levels in males **(d)** and females **(l)** at 8 weeks. Body composition analysis: fat mass **(e, m)**, lean mass **(f, n)**, fat-to-body ratio **(g, o)**, and lean-to-body ratio **(h, p)** in Mut (Sal) vs. Mut (GH). Data: Mean ± SEM (n = 6-8). Statistical significance: two-way ANOVA for growth curves, t-test for other comparisons (*p < 0.05, **p < 0.01, ***p < 0.001, ****p < 0.0001). Body composition analysis was presented as mean ± SEM, n = 6 per group, and statistical significance was determined using an unpaired t-test followed by the Mann-Whitney test. Significance levels are indicated as follows: * p < 0.05, **p < 0.01, ***p < 0.001, ****p < 0.0001.

In male Mut mice, a notable effect of GH treatment on BW (**Figure 3a**) becomes significant (p < 0.0014) at 6 weeks of age (corresponding to week 2 of treatment), and this difference remains significant (p < 0.0001) until 8 weeks of age (corresponding to 4 weeks of GH treatment). Specifically, male Mut mice treated with GH for 4 weeks exhibited a significant increase in BW (13.47 ± 0.76 g) compared to Mut control (10.25 ± 0.308 g). Additionally, BL in male Mut mice (**Figure 3b**) showed a significant (p < 0.05) difference starting at week 5 of age (after 1 week of GH treatment) and continued until 8 weeks (p < 0.0001) of age compared to the control group. Male Mut mice treated with GH for 4 weeks displayed a significant increase in BL (6.37 ± 0.15 cm) compared to Mut control (7.5 ± 0.21) cm.

Overall, similar patterns were observed in female Mut mice as the effect of GH treatment on BW (**Figure 3i**) started to become significant (p < 0.005) at week 7 of age and persisted until the end of the study. Specifically, female Mut mice treated with GH for 4 weeks exhibited a significant increase in BW (10.51 ± 0.7 g) compared to Mut control (8.25 ± 0.96 g). BL in female Mut mice (**Figure 3j**) started to become significant (p < 0.05) at week 6 of age and continued until week 8 of age. Female Mut mice treated with GH for 4 weeks displayed a significant increase (p <0.001) in BL (6.75 ± 0.095) cm compared to the Mut control (6.12 ± 0.065 cm). Representative images of male (**Figure 3c**) and female (**Figure 3k**) Mut mice treated with saline or GH further illustrate these differences.

As expected, the baseline IGF-I serum levels in both male (**Figure 3d**) and female (**Figure 3l**) WT mice were significantly higher compared to Mut mice treated with either saline or GH (p < 0.0001). In males, WT mice exhibited an IGF-I serum level of 268.21 ± 7.413 ng/mL, significantly higher than that observed in Mut mice treated with saline (95.3 ± 6.58 ng/mL) and Mut mice treated with GH (158.47 ± 6.16 ng/mL). Similarly, in females, WT mice had an IGF-I serum level of 306.51 ± 8.43 ng/mL, compared to 102.03 ± 6.71 ng/mL in saline-treated Mut mice and 183.32 ± 7.86 ng/mL in GH-treated Mut mice. It is worth noting that, despite the significant elevation in serum IGF-I levels observed in Mut mice treated with GH, full restoration of normal IGF-I levels, as seen in WT mice, was not achieved.

Given the observed changes in growth, we conducted an EchoMRI analysis to investigate the effects of GH treatment on body composition. In male Mut mice (**Figures 3e-h**), GH treatment caused a significant (p = 0.028) reduction in total fat mass (**Figure 3e**) and a significant (p = 0.027) increase in total lean mass (**Figure 3f**) compared to Mut control mice. Specifically, the fat mass in mice treated with saline was 2.45 ± 0.12 g, while in GH-treated mice, it was significantly lower at 1.4 ± 0.006 g. The lean mass in Mut control mice was 7.89 ± 0.3 g, whereas GH-treated mice exhibited a lean mass of 11.25 ± 0.064 g. Furthermore, GH treatment significantly affected the body composition ratios. The fat/body weight ratio (**Figure 3g**) decreased from 22.68 ± 1.34% in Mut control mice to 15.86 ± 0.024% in GH-treated mice (p = 0.02). On the other hand, the lean/body weight ratio (**Figure 3h**) increased from 75.56 ± 0.341% in Mut control mice to 84.13 ± 0.264% in GH-treated mice (p = 0.05).

In female Mut mice (**Figures 3m-p**), GH treatment had similar effects as those observed in male Mut mice. A significant (p = 0.006) reduction in fat mass was observed (**Figure 3m**). Specifically, female Mut control mice had a fat mass of 2.11 ± 0.09 g, while those treated with GH had a significantly lower fat mass of 1.179 ± 0.061 g. Regarding lean mass (**Figure 3n**), there was a significant (p < 0.029) increase in GH-treated mice. Female Mut control had a lean mass of 6.948 ± 0.134 g, while GH-treated mice had a lean mass of 9.340 ± 0.215 g. GH treatment also significantly affected body composition ratios in female mice. The fat/body weight ratio (**Figure 3o**) showed a significant reduction (p < 0.027), with Mut control mice having a ratio of 22.105 ± 1.56%, while GH-treated mice had a reduced ratio of 11.68 ± 0.87%. On the other hand, the lean/body weight ratio (**Figure 3p**) increased significantly (p < 0.05) from 77.89 ± 1.5% in Mut control mice to 88.32 ± 0.78% in GH-treated mice.

### Assessment of metabolic effect of GH treatment on the Pit-1^^K216E^ mice

The effects of GH treatment on metabolic parameters were evaluated using the CLAMS in both male and female Mut mice at 8 weeks of age. GH treatment was initiated at 4 weeks of age and continued for 4 weeks, with a separate group of Mut mice receiving saline instead of GH as a control.

**Figure 4:**
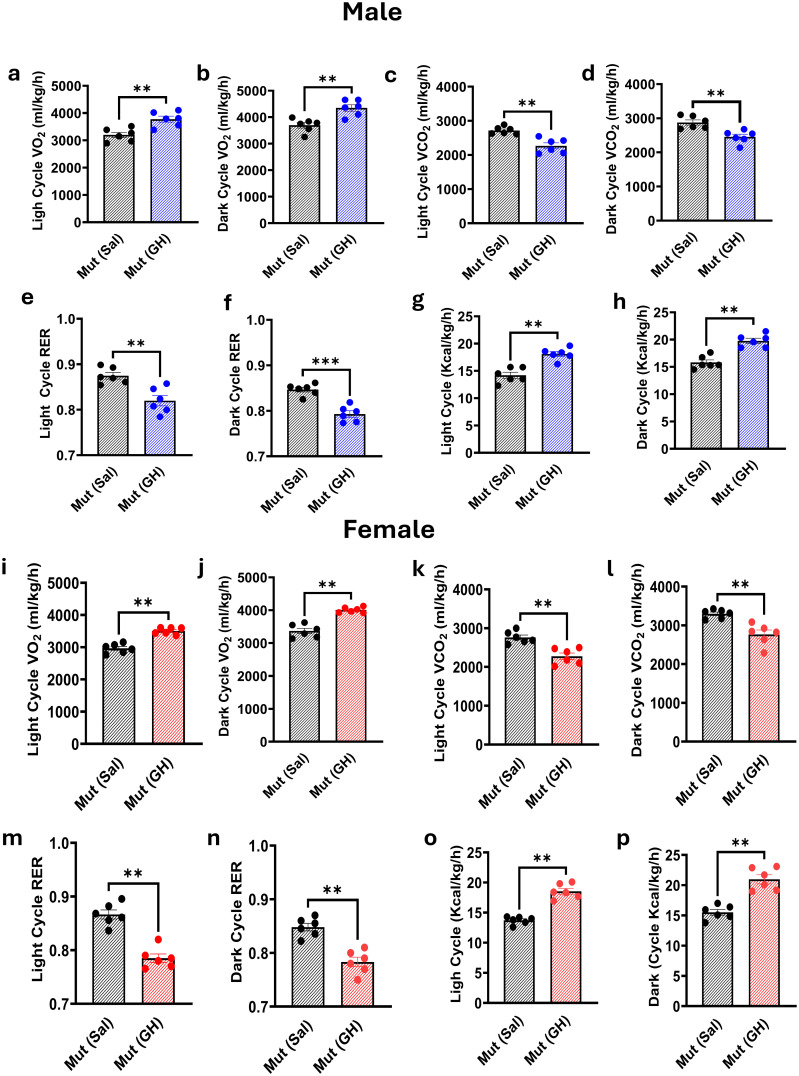
Effect of GH Treatment on the Metabolic Profile of Pit-1^^K216E^ Mutant Mice. This figure illustrates the impact of GH treatment on metabolic parameters in Mut mice at 13 weeks post-GH treatment. Males: VO_2_ **(a, b)**, VCO_2_ **(c, d)**, RER **(e, f)**, and energy expenditure **(g, h)** during light and dark cycles. Females: VO_2_ **(i, j)**, VCO_2_ **(k, l)**, RER **(m, n)**, and energy expenditure **(o, p)** during light and dark cycles. Data: Mean ± SEM (n = 6). Results are presented as mean ± SEM, n=6. Statistical analysis was performed using an unpaired t-test followed by the Mann-Whitney test. n=6 Significance is indicated as follows: ns (not significant), * p<0.05, ** p<0.01, *** p < 0.001.

In male Mut mice, GH treatment resulted in significant changes in several metabolic parameters compared to the control group. Specifically, GH-treated male mice exhibited a significant increase in oxygen consumption (VO_2_) during both the light (3778.33 ± 110.16 ml/kg/h, p < 0.05; **Figure 4a**) and dark (4349.33 ± 123.09 ml/kg/h, p < 0.043; **Figure 4b**) cycles compared to Mut controls (Light: 3198.88 ± 98.17 ml/kg/h, Dark: 3696.01 ± 104.89 ml/kg/h). Additionally, GH-treated male mice showed a significant decrease in carbon dioxide production (VCO_2_) during both the light (2268.19 ± 86.94 ml/kg/h, p < 0.0021; **Figure 4c**) and dark (2455.52 ± 76.52 ml/kg/h, p = 0.0035; **Figure 4d**) cycles compared to saline-treated controls (Light: 2718.14 ± 44.00 ml/kg/h, Dark: 2926.34 ± 74.39 ml/kg/h). The respiratory exchange ratio (RER) was also significantly reduced in GH-treated male mice during both the light (0.820 ± 0.011, p = 0.0045; **Figure 4e**) and dark (0.793 ± 0.007, p = 0.0001; **Figure 4f**) cycles compared to saline-treated controls (Light: 0.875 ± 0.007, Dark: 0.847 ± 0.005). Furthermore, GH-treated male mice exhibited a significant increase in heat expenditure during both the light (18.09 ± 0.45 Kcal/kg/hr, p < 0.002; **Figure 4g**) and dark (19.78 ± 0.48 Kcal/kg/hr, p < 0.003; **Figure 4h**) cycles compared to saline-treated controls (Light: 14.20 ± 0.55 Kcal/kg/hr, Dark: 15.82 ± 0.47 Kcal/kg/hr).

Similarly, in female Mut mice, GH treatment led to significant changes in metabolic parameters. GH-treated female mice showed significantly higher VO_2_ during both the light (3509.40 ± 41.72 ml/kg/h, p = 0.002; **Figure 4i**) and dark (4006.14 ± 33.62 ml/kg/h, p = 0.004; **Figure 4j**) cycles compared to saline-treated controls (Light: 2966.80 ± 58.79 ml/kg/h, Dark: 3367.23 ± 81.14 ml/kg/h). In contrast, VCO_2_ was significantly reduced in GH-treated female mice during both the light (2275.37 ± 83.71 ml/kg/h, p = 0.002; **Figure 4k**) and dark (2765.28 ± 113.11 ml/kg/h, p = 0.004; **Figure 4l**) cycles compared to saline-treated controls (Light: 2759.83 ± 63.12 ml/kg/h, Dark: 3297.69 ± 46.68 ml/kg/h). The RER was significantly lower in GH-treated female mice during both the light (0.785 ± 0.008, p = 0.0034; **Figure 4m**) and dark (0.783 ± 0.009, p = 0.0035; **Figure 4n**) cycles compared to saline-treated controls (Light: 0.867 ± 0.008, Dark: 0.848 ± 0.007). Moreover, GH-treated female mice exhibited a significant increase in heat production during both the light (18.54 ± 0.50 Kcal/kg/hr, p = 0.003; **Figure 4o**) and dark (20.98 ± 0.75 Kcal/kg/hr, p = 0.002; **Figure 4p**) cycles compared to saline-treated controls (Light: 13.72 ± 0.26 Kcal/kg/hr, Dark: 15.51 ± 0.48 Kcal/kg/hr).

### Metabolomic profiling and biomarkers identification of Pit-1^^K216E^ mouse model

**Figure 5:**
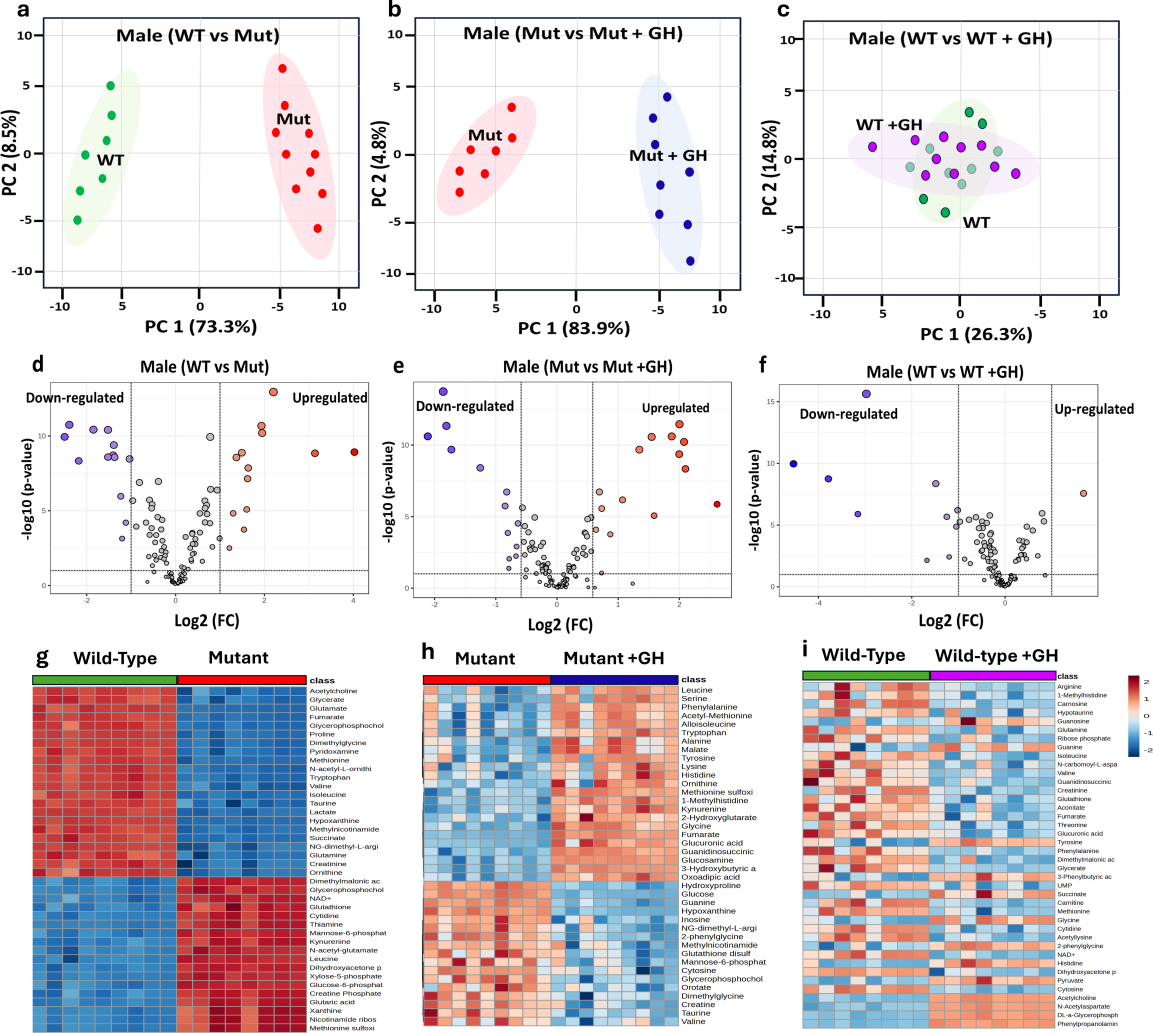
Comparative metabolomic analysis to identify GHD Biomarkers in male mice. This figure examines metabolomic profiles under different GH conditions to identify GHD biomarkers and GH treatment effects. PCA plots **(a–c)** show metabolic distinctions: **(a)** GH sufficient (WT) vs. GHD (Mut), **(b)** GHD (Mut) vs. GH supplemented (Mut + GH), and **(c)** GH sufficient (WT) vs. GH excess (WT + GH). Volcano plots **(d–f)** display differentially expressed metabolites based on Log2 fold change and p-values for the same comparisons. Heatmaps **(g–i)** illustrate key metabolite expression across groups.

To identify metabolites that could potentially serve as biomarkers for the GHD condition and monitor the efficacy of GH treatment in GHD, we first examined the differences in metabolomic profiles between WT and Mut mice. In this study, we employed untargeted LC-MS combined with univariate statistical analysis to explore the differences in metabolomic profiles (metabolites) across distinct conditions. In males, the PCA demonstrated clear separation between WT (green dot) and Mut (red dots) mice at 8 weeks of age along principal component 1 (PC1) and principal component 2 (PC2), explaining 73.3% and 8.5% of the variance, respectively (**Figure 5a**). The PCA in (**Figure 5b**) compares the metabolomic profiles of Mut control (saline-treated) mice versus Mut mice treated with GH for 4 weeks (blue dots) illustrating the metabolic changes induced by GH treatment in GHD. The analysis highlights the effect of GH treatment on the GHD mouse model, with PC1 accounting for 83.9% of the variance and PC2 accounting for 4.8%. Furthermore, the PCA compared the metabolomic profiles of WT control mice to WT treated with GH (purple dots). The results show no clear separation between the two groups. The overlapping clusters indicate that the metabolic pathways remain largely similar in both control and GH-treated WT mice, with PC1 accounting for 26.3% of the variance and PC2 accounting for 14.8% (**Figure 5c**).

The volcano plots in (**Figures 5d-f**) further support these findings, revealing significant upregulation and downregulation of numerous metabolites in WT compared to Mut mice. GH treatment in Mut mice led to notable changes in metabolite expression, with many metabolites showing significant shifts towards normalization (**Figure 5e**). In contrast, GH treatment in WT mice resulted in relatively few significant changes in metabolite expression (**Figure 5f**). The heat maps in **Figures 5g-i** provide a detailed visualization of the metabolite changes across different groups of male mice, supporting the findings from the PCA and volcano plots.

**Figure 5g** highlights clear differences in metabolite expression between WT and Mut mice, with distinct patterns of upregulation and downregulation. **Figure 5h** shows that GH treatment in GHD Mut mice leads to a partial normalization of these metabolites. In contrast, **Figure 5i** reveals minimal changes in metabolite expression between control and GH-treated WT mice.

Furthermore, we employed ROC analysis to identify signature biomarkers specific to multiple conditions. Metabolites identified as specific for GHD through a comparison of WT versus Mut mice were termed Baseline GHD Biomarkers, highlighting the metabolomic alterations associated with GHD. These biomarkers exhibited significant log2 fold changes (Log2 FC), isuch as acetylcholine (6.75), creatine phosphate (-7.22), creatinine (4.89), cytidine (-4.10), cytosine (2.30), glucose-6-phosphate (-6.39), glucuronic acid (3.30), glutamine (3.67), glutamic acid (-6.67), glutathione (-3.91), hypotaurine (-2.79), lactate (12.33), proline (-14.11), pyridoxamine (6.64), ribose phosphate (-3.15), taurine (4.2) and uric acid (4.08) (**Table 1**).

**Table 1:** Comparative Analysis of Biomarkers Across GH Sufficient, GH Deficient, and GH Response Conditions in WT and Mut Male Mice.

Biomarkers	Wild-type vs Mutant (Male)						Mutant vs Mutant (GH) (Male)						Wild vs Wild type (GH) (Male)					
	Metabolites	AUC	*(p)* Value	Log 2 (FC)	± SD	Cohen’s d	Metabolites	AUC	*(p)* Value	Log 2 (FC)	± SD	Cohen’s d	Metabolites	AUC	*(p)* Value	Log 2 (FC)	± SD	Cohen’s d
GHD biomarkers	Acetylcholine	1.0	0.000007	6.75	1.7	3.97	Acetylcholine	0.8	0.198186	0.17	0.23	0.76	Acetylcholine	0.6	0.987000	0.96	0.56	1.714
	Creatine Phosphate	1.0	0.000008	-7.22	1.2	-6.02	Creatine Phosphate	0.6	0.553727	0.14	0.27	0.53	Creatine Phosphate	0.6	0.454183	0.14	0.18	0.796
	Creatinine	1.0	0.000012	4.89	1.32	3.71	Creatinine	0.6	0.554043	0.09	0.17	0.55	Creatinine	0.8	0.136889	-0.27	0.23	-1.183
	Cytidine	1.0	0.000001	-4.10	1.02	-4.02	Cytidine	0.8	0.190080	0.28	0.37	0.77	Cytidine	0.9	0.021049	-0.30	0.12	-2.494
	Cytosine	1.0	0.000280	2.30	0.74	3.11	Cytosine	1.0	0.026418	0.42	0.61	0.69	Cytosine	1.0	0.001567	-0.32	0.41	-0.776
	Glucose-6-phosphate	1.0	0.000002	-6.39	1.53	-4.18	Glucose-6-phosphate	0.8	0.370925	0.40	0.55	0.73	Glucose-6-phosphate	0.6	0.390560	0.20	0.13	1.565
	Glucuronic acid	1.0	0.000060	3.30	1.13	2.92	Glucuronic acid	1.0	0.004025	-1.29	0.78	-1.66	Glucuronic acid	0.9	0.040278	-0.15	0.09	-1.704
	Glutamine	1.0	0.000008	3.76	1.24	3.03	Glutamine	0.8	0.138111	-0.25	0.42	-0.60	Glutamine	0.8	0.226548	0.03	0.07	0.431
	Glutaric acid	1.0	0.000001	-6.67	1.54	-4.33	Glutaric acid	0.6	0.640495	0.29	0.36	0.81	Glutaric acid	0.5	0.583453	0.11	0.07	1.550
	Glutathione	1.0	0.000022	-3.91	1.47	-2.66	Glutathione	0.7	0.880591	-0.06	0.24	-0.24	Glutathione	0.8	0.109659	-0.50	0.14	-3.546
	Hypotaurine	1.0	0.000028	-2.79	0.78	-3.58	Hypotaurine	0.6	0.570489	0.17	0.19	0.88	Hypotaurine	0.9	0.152481	0.10	0.08	1.238
	Lactate	1.0	0.000002	12.33	2.1	5.87	Lactate	0.6	0.424572	0.10	0.09	1.15	Lactate	0.6	0.997899	0.33	0.12	2.762
	Proline	1.0	0.000345	13.04	1.78	7.33	Proline	0.6	0.577598	0.05	0.09	0.56	Proline	0.6	0.553560	-0.07	0.03	-2.249
	Purine	1.0	0.000006	-14.11	1.87	-7.55	Purine	0.8	0.127130	0.65	0.73	0.89	Purine	0.5	0.921915	0.16	0.04	4.490
	Pyridoxamine	1.0	0.000001	6.64	1.21	5.49	Pyridoxamine	0.7	0.412279	0.07	0.02	3.48	Pyridoxamine	0.7	0.421105	0.77	0.13	5.900
	Ribose phosphate	1.0	0.000017	-3.15	0.87	-3.63	Ribose phosphate	0.8	0.641095	-0.24	0.17	-1.44	Ribose phosphate	0.8	0.163197	-0.06	0.04	-1.491
	Taurine	1.0	0.000011	4.20	1.16	3.62	Taurine	1.0	0.009437	0.33	0.21	1.57	Taurine	0.6	0.760572	0.44	0.21	2.088
	Uric acid	1.0	0.000159	4.08	1.3	3.14	Uric acid	0.6	0.995515	0.01	0.00	3.42	Uric acid	0.7	0.242496	-0.78	0.44	-1.765
GH Treatment Responsive Biomarkers	3-Hydroxybutyric acid	1.0	0.010290	2.75	1.25	2.20	3-Hydroxybutyric acid	1.0	0.000188	-2.51	0.98	-2.56	3-Hydroxybutyric acid	0.6	0.654986	0.18	0.36	0.495
	AMP	1.0	0.000047	3.15	1.26	2.50	AMP	0.9	0.023798	-1.90	1.02	-1.86	AMP	0.7	0.383062	-0.05	0.03	-1.595
	Fumarate	1.0	0.000440	5.94	1.27	4.68	Fumarate	1.0	0.000008	-2.05	1.07	-1.92	Fumarate	0.8	0.172170	-0.06	0.04	-1.535
	Glucose	1.0	0.003632	-4.84	1.37	-3.53	Glucose	1.0	0.000024	2.41	1.10	2.19	Glucose	0.9	0.054286	-0.20	0.80	-0.247
	Glutamate	1.0	0.000029	6.40	1.54	4.15	Glutamate	1.0	0.006130	1.54	0.97	1.59	Glutamate	0.8	0.421595	0.56	0.33	1.693
	Glycine	1.0	0.000317	2.30	1.3	1.77	Glycine	1.0	0.002455	-1.71	0.80	-2.14	Glycine	0.6	0.747979	0.26	0.14	1.883
	Guanine	1.0	0.000158	2.63	1.31	2.01	Guanine	1.0	0.000724	2.14	0.45	4.76	Guanine	0.9	0.048794	1.04	0.88	1.177
	Hydroxyproline	1.0	0.000042	-3.12	1.32	-2.36	Hydroxyproline	1.0	0.000299	2.10	0.67	3.14	Hydroxyproline	1.0	0.010500	-0.37	0.24	-1.533
	Hypoxanthine	1.0	0.000029	10.71	1.87	5.73	Hypoxanthine	1.0	0.000161	1.62	0.54	2.99	Hypoxanthine	0.8	0.154208	0.68	0.33	2.063
	Kynurenine	1.0	0.000002	-5.62	1.23	-4.57	Kynurenine	1.0	0.012258	-1.84	0.47	-3.91	Kynurenine	0.5	0.984855	0.30	0.17	1.759
	Leucine	1.0	0.000005	3.92	0.98	4.00	Leucine	0.8	0.006546	-1.65	0.51	-3.24	Leucine	0.6	0.548160	0.25	0.10	2.510
	Methionine	1.0	0.000067	9.25	1.36	6.80	Methionine	1.0	0.004508	2.11	0.33	6.39	Methionine	0.9	0.060732	-0.44	0.60	-0.738
	Methionine sulfoxide	1.0	0.000002	7.00	1.22	5.73	Methionine sulfoxide	1.0	0.002795	-2.63	0.67	-3.92	Methionine sulfoxide	0.5	0.933426	0.16	0.12	1.316
	Pyruvate	1.0	0.000095	4.11	1.3	3.16	Pyruvate	0.9	0.006527	-2.22	0.70	-3.17	Pyruvate	1.0	0.004812	1.00	0.56	1.793
	Tryptophan	1.0	0.000000	5.58	0.99	5.64	Tryptophan	0.9	0.036891	-1.84	0.40	-4.60	Tryptophan	0.6	0.515975	0.24	0.60	0.404
	Tyrosine	0.9	0.016837	2.04	0.78	2.62	Tyrosine	0.9	0.008798	-1.52	0.35	-4.33	Tyrosine	0.6	0.635533	0.43	0.10	4.269
	Xanthine	1.0	0.000017	-4.60	0.87	-5.29	Xanthine	0.8	0.001423	-2.15	0.42	-5.12	Xanthine	0.5	0.669474	0.06	0.04	1.420
	Xylose-5-phosphate	1.0	0.000001	-3.90	1.2	-3.25	Xylose-5-phosphate	1.0	0.002934	-2.36	0.77	-3.06	Xylose-5-phosphate	0.6	0.582535	0.23	0.14	1.662
GH Treatment-Specific Responsive Biomarkers	Glutathione disulfide	0.9	0.015295	0.71	0.72	0.98	Glutathione disulfide	0.9	0.044909	1.65	0.65	2.53	Glutathione disulfide	0.5	0.659690	0.18	0.11	1.636
	Glucosamine	1.0	0.014066	0.07	0.23	0.30	Glucosamine	1.0	0.000710	-2.47	1.00	-2.47	Glucosamine	0.7	0.367665	0.49	0.31	1.571
	Guanidinosuccinic acid	1.0	0.000071	0.26	0.33	0.79	Guanidinosuccinic acid	1.0	0.001484	-1.67	0.87	-1.91	Guanidinosuccinic acid	0.8	0.120885	-0.18	0.08	-2.348
	Guanosine	1.0	0.003945	-0.73	0.87	-0.84	Guanosine	1.0	0.006188	-2.22	0.49	-4.54	Guanosine	0.9	0.047164	1.05	0.87	1.205

Biomarker analysis comparing WT vs. Mut (GHD), Mutant (GHD) vs. Mutant (GH), and WT vs. WT (GH). Metabolites are categorized as GHD Biomarkers, GH treatment-responsive biomarkers, and GH treatment-specific responsive Biomarkers. The table includes AUC, *p*-value, and Log_2_FC.

Additionally, a subset of metabolites, termed GH Treatment Responsive Biomarkers, exhibited significant log2 FC, indicating their relevance in both the GHD condition and the response to GH treatment in GHD. This group includes 3-hydroxybutyric acid (2.75), AMP (3.15), fumarate (5.94), glucose (-4.84), glutamate (6.40), glycine (2.30), guanine (2.63), hydroxyproline (-3.12), hypoxanthine (10.71), kynurenine (-5.62), leucine (3.92), methionine (9.25), Methionine sulfoxide (7.0) pyruvate (4.11), tryptophan (5.58), tyrosine (2.04), xanthine (-4.60), xylose-5-phosphate (-3.90) (**Table 1**). Further analysis identified a GH Treatment-Specific Responsive Biomarkers, which exhibited significant log2 FC in response to GH treatment only. This group includes glutathione disulfide (1.65), glucosamine (-2.47), guanidinosuccinic acid (-1.67), and guanosine (-2.22). These potential biomarkers demonstrated significant p-values (<0.005) and high AUC values (>0.9), underscoring their strong association with the condition and their potential as reliable indicators (**Table 1**).

**Figure 6:**
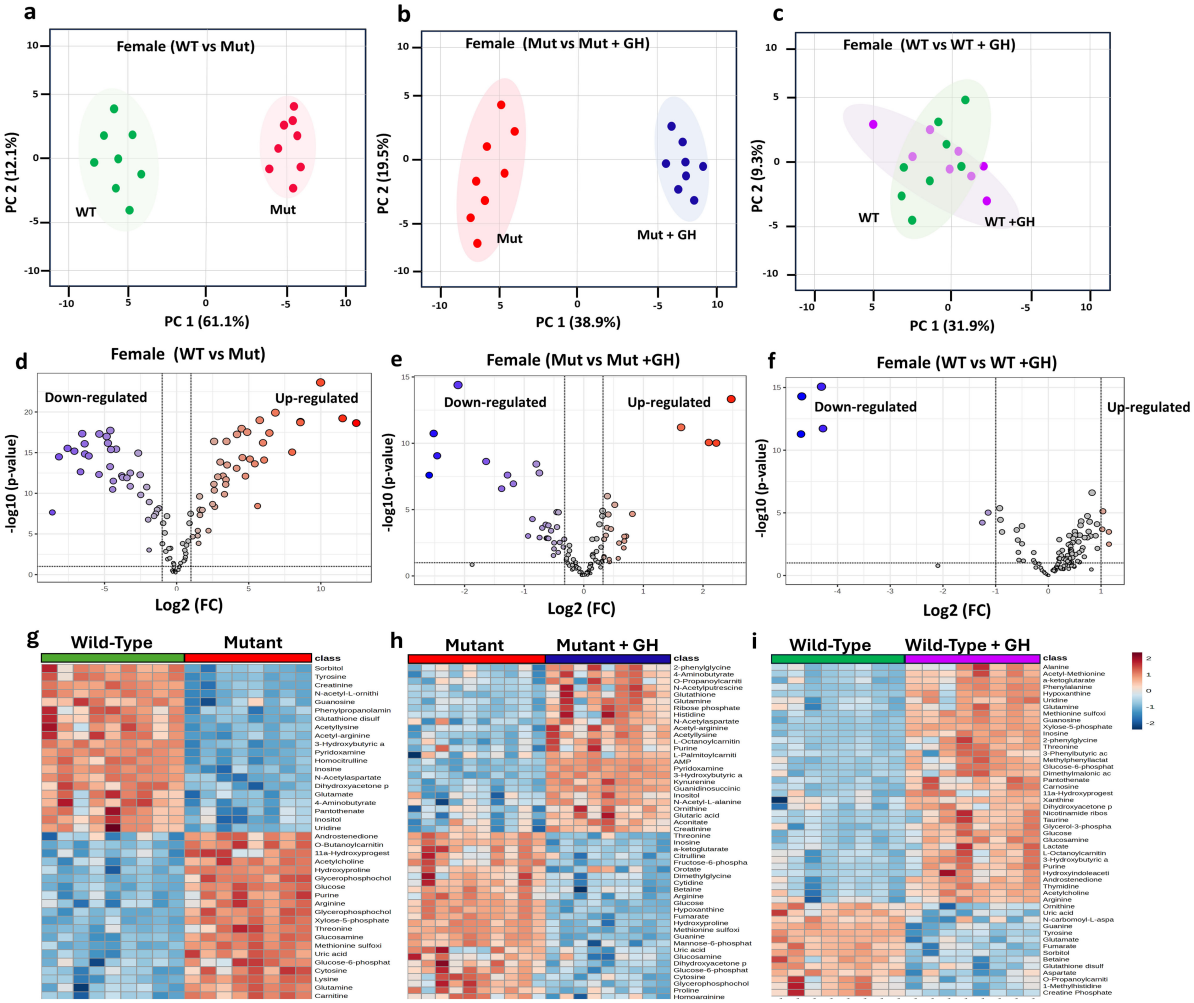
Comparative Metabolomic Analysis to Identify GHD Biomarkers in Female Mice. This figure analyzes metabolomic profiles under different GH conditions to identify GHD biomarkers and GH treatment effects. PCA plots **(a-c)** show metabolic distinctions: **(a)** GH sufficient (WT) vs. GHD (Mut), **(b)** GHD (Mut) vs. GH supplemented (Mut + GH), and **(c)** GH sufficient (WT) vs. GH excess (WT + GH), with **(c)** showing no clear separation. Volcano plots **(d-f)** highlight differentially expressed metabolites based on Log2 fold change and p-values for the same comparisons. Heatmaps **(g-i)** illustrate key metabolite expression across groups.

In females, the PCA demonstrated a clear separation between WT (green dot) and Mut (red dots) mice at 8 weeks of age along PC1 and PC2, explaining 61.1% and 12.1% of the variance, respectively **Figure 6a**. The PC analysis in **Figure 6b** compares the metabolomic profiles of Mut mice versus Mut mice treated with GH for 4 weeks (blue dots) illustrating the metabolic changes induced by GH treatment in GHD. The analysis highlights the effect of GH treatment on the GHD mouse model, with the PC1 accounting for 38.9.9% of the variance and the PC2 accounting for 19.5%. The distinct separation between the two groups highlights the significant metabolic shifts induced by GH treatment in the Mut mice. Furthermore, the PCA compares the metabolomic profiles of WT mice to WT treated with GH (purple dots). The results show no clear separation between the two groups. The overlapping clusters indicate that the metabolic pathways remain largely similar in both control and GH-treated mice, with PC1 accounting for 31.9% of the variance and PC2 accounting for 9.3%. The volcano plots in **Figures 6d-f** further support these findings, revealing significant upregulation and downregulation of numerous metabolites in WT mice compared to Mut. GH treatment in Mut mice led to notable changes in metabolite expression, with many metabolites showing significant shifts towards normalization **Figure 6e**. In contrast, GH treatment in WT mice resulted in relatively few significant changes in metabolite expression **Figure 6f**. The heat maps in **Figures 6g–i** provide a detailed visualization of the metabolic changes across different groups of female mice, supporting the findings from the PCA and volcano plots. **Figure 6g** highlights clear differences in metabolite expression between WT and Mut mice, with distinct patterns of upregulation and downregulation, indicating significant metabolic alterations associated with the GHD condition. **Figure 6h** shows that GH treatment in GHD Mut mice leads to a partial normalization of these metabolic disruptions, with many metabolites shifting towards expression levels similar to those in WT mice. In contrast, **Figure 6i** reveals minimal changes in metabolite expression between control and GH-treated WT mice, highlighting that GH treatment has a more pronounced effect in restoring metabolic balance in GHD conditions compared to its impact on the metabolic profiles in GH-sufficient WT mice.

In females, the GHD Biomarkers highlight the metabolomic alterations associated with GHD. These biomarkers exhibited significant Log2 FC, including glucosamine (-1.92), homocitrulline (2.16), lysine (-1.95), N-Acetylaspartate (1.75), N-acetyl-L-ornithine (2.31), N-carbomoyl-L-aspartate (1.75), phenylpropanolamine (2.14), purine (-2.82), and sorbitol (1.99). These metabolites were found to be significantly altered in GHD conditions, reflecting the metabolic shifts occurring in response to this condition in female mice (**Table 2**).

**Table 2:** Comparative Analysis of Biomarkers Across GH Sufficient, GH Deficient, and GH Response Conditions in WT and Mut Female Mice.

Biomarkers	Wild-type vs Mutant (Female)						Mutant vs Mutant (GH) (Female)						Wild-type vs Wild-type (GH) (Female)					
	Metabolites	AUC	*(p)* Value	Log 2 (FC)	± SD	Cohen’s d	Metabolites	AUC	*(p)* Value	Log 2 (FC)	± SD	Cohen’s d	Metabolites	AUC	*(p)* Value	Log 2 (FC)	± SD	Cohen’s d
GHD biomarkers	Glucosamine	1.0	0.000008	-1.92	0.66	-2.91	Glucosamine	0.9	0.032723	0.53	0.32	1.66	Glucosamine	1.0	0.003559	-0.16	0.11	-1.459273
	Homocitrulline	1.0	0.000007	2.16	0.78	2.77	Homocitrulline	0.5	0.814281	0.11	0.10	1.15	Homocitrulline	0.6	0.593923	0.38	0.33	1.1545152
	Lysine	1.0	0.000002	-1.95	0.67	-2.91	Lysine	0.8	0.138610	0.47	0.21	2.24	Lysine	1.0	0.146423	-0.47	0.12	-3.943167
	N-Acetylaspartate	1.0	0.000069	1.75	1.10	1.59	N-Acetylaspartate	0.8	0.094748	-0.18	0.14	-1.28	N-Acetylaspartate	0.6	0.404481	0.35	0.1	3.5443
	N-acetyl-L-ornithine	1.0	0.000012	2.31	1.00	2.31	N-acetyl-L-ornithine	0.6	0.596978	0.37	0.16	2.37	N-acetyl-L-ornithine	0.5	0.846506	0.17	0.22	0.7697273
	N-carbomoyl-L-aspartate	0.9	0.042521	1.75	1.02	1.71	N-carbomoyl-L-aspartate	0.6	0.433769	-0.44	0.20	-2.22	N-carbomoyl-L-aspartate	1.0	0.000420	1.05	0.43	2.4493023
	Phenylpropanolamine	1.0	0.001632	2.14	0.94	2.27	Phenylpropanolamine	0.8	0.180769	-0.50	0.33	-1.53	Phenylpropanolamine	1.0	0.011525	-0.54	0.33	-1.629152
	Purine	1.0	0.000057	-2.82	0.71	-3.97	Purine	0.9	0.143366	-0.04	0.45	-0.09	Purine	0.6	0.690727	0.24	0.12	2.0290833
	Sorbitol	1.0	0.000410	1.99	0.48	4.15	Sorbitol	0.8	0.079770	-0.62	0.43	-1.44	Sorbitol	0.6	0.404481	0.35	0.3	1.1814333
GH Treatment Responsive Biomarkers	3-Hydroxybutyric acid	1.0	0.000027	2.37	0.88	2.69	3-Hydroxybutyric acid	1.0	0.000018	-2.99	0.65	-4.59	3-Hydroxybutyric acid	0.7	0.712829	0.13	0.15	0.8396
	AMP	0.9	0.009500	1.87	0.42	4.46	AMP	1.0	0.000009	-1.72	0.98	-1.76	AMP	0.9	0.013216	0.09	0.1	0.924
	Fumarate	1.0	0.000003	2.44	1.30	1.88	Fumarate	1.0	0.000073	2.22	1.10	2.02	Fumarate	0.5	0.955457	0.18	0.4	0.46135
	Glucose	1.0	0.000013	-2.16	1.10	-1.96	Glucose	1.0	0.000046	2.14	1.30	1.64	Glucose	0.7	0.297244	0.11	0.2	0.54415
	Glutamate	1.0	0.004105	1.70	0.69	2.47	Glutamate	0.9	0.009228	2.11	1.32	1.60	Glutamate	1.0	0.003908	0.74	0.53	1.3909811
	Glutathione disulfide	1.0	0.000001	2.76	1.30	2.12	Glutathione disulfide	1.0	0.007359	1.72	1.10	1.57	Glutathione disulfide	0.8	0.139327	0.45	0.6	0.7564667
	Glycine	1.0	0.040534	1.80	1.10	1.64	Glycine	0.8	0.019816	-1.80	1.20	-1.50	Glycine	0.6	0.732173	0.26	0.8	0.322075
	Guanidinosuccinic acid	1.0	0.022028	1.85	1.21	1.53	Guanidinosuccinic acid	1.0	0.000027	-1.58	1.10	-1.43	Guanidinosuccinic acid	0.8	0.778106	-0.03	0.02	-1.5356
	Guanine	1.0	0.001491	2.04	0.98	2.08	Guanine	1.0	0.000017	1.99	0.87	2.29	Guanine	1.0	0.002017	1.18	0.54	2.1940741
	Guanosine	1.0	0.006180	1.70	0.63	2.69	Guanosine	0.9	0.037096	-1.51	1.11	-1.36	Guanosine	1.0	0.000031	-0.18	0.33	-0.549394
	Hydroxyproline	1.0	0.000006	-1.85	0.55	-3.36	Hydroxyproline	1.0	0.000230	2.33	0.87	2.67	Hydroxyproline	0.7	0.823236	0.32	0.6	0.5263
	Hypoxanthine	1.0	0.002220	2.65	1.30	2.04	Hypoxanthine	1.0	0.000000	2.84	0.77	3.68	Hypoxanthine	1.0	0.000033	1.01	0.44	2.2929545
	Inosine	1.0	0.000032	3.66	1.54	2.38	Inosine	1.0	0.000052	1.67	0.99	1.68	Inosine	1.0	0.000123	-1.34	0.65	-2.064154
	Kynurenine	0.8	0.000678	-2.84	1.33	-2.14	Kynurenine	1.0	0.000250	-2.11	0.74	-2.85	Kynurenine	0.6	0.873483	0.18	0.12	1.4983333
	Leucine	0.9	0.005294	1.83	0.77	2.38	Leucine	0.8	0.039876	-2.01	0.77	-2.60	Leucine	0.7	0.724244	0.32	0.45	0.7045556
	Methionine	0.9	0.004890	3.76	0.73	5.15	Methionine	1.0	0.007066	1.93	0.93	2.08	Methionine	0.5	0.978685	0.22	0.6	0.3709667
	Methionine sulfoxide	1.0	0.000036	2.35	1.30	1.81	Methionine sulfoxide	1.0	0.000005	2.22	0.74	2.99	Methionine sulfoxide	1.0	0.026434	1.12	0.2	5.5835
	Pyridoxamine	1.0	0.000005	2.29	1.30	1.76	Pyridoxamine	1.0	0.000001	-2.68	0.88	-3.05	Pyridoxamine	0.5	0.978685	0.22	0.6	0.3709667
	Pyruvate	0.9	0.022555	1.96	1.22	1.60	Pyruvate	1.0	0.005263	1.72	0.74	2.32	Pyruvate	1.0	0.026434	1.12	0.4	2.79175
	Tryptophan	0.9	0.046564	1.52	1.77	0.86	Tryptophan	1.0	0.030955	1.83	0.63	2.91	Tryptophan	0.5	0.846506	0.17	0.2	0.8467
	Tyrosine	1.0	0.000060	4.46	1.70	2.62	Tyrosine*	0.8	0.007890	2.77	0.99	2.80	Tyrosine	0.9	0.026671	1.13	0.4	2.8255
	Xanthine	1.0	0.000554	-1.67	1.10	-1.52	Xanthine*	0.8	0.003290	-1.94	1.10	-1.76	Xanthine	0.6	0.803531	0.26	0.15	1.7312667
	Xylose-5-phosphate	1.0	0.000006	-3.37	1.30	-2.59	Xylose-5-phosphate	1.0	0.009970	-2.37	1.33	-1.78	Xylose-5-phosphate	0.6	0.939908	0.21	0.44	0.4795
GH Treatment-Specific Responsive Biomarkers	a-ketoglutarate	0.6	0.564191	0.32	0.34	0.95	a-ketoglutarate	1.0	0.005019	1.86	1.02	1.82	a-ketoglutarate	1.0	0.010213	-0.24	0.26	-0.931115
	Fructose-6-phosphate	0.6	0.550131	0.16	0.12	1.33	Fructose-6-phosphate	1.0	0.002674	2.71	1.30	2.09	Fructose-6-phosphate	0.6	0.707040	0.08	0.09	0.8470778
	Glucose-6-phosphate	0.9	0.011616	-0.29	0.31	-0.94	Glucose-6-phosphate	1.0	0.001717	1.72	0.78	2.20	Glucose-6-phosphate	1.0	0.009384	-0.46	0.83	-0.557193
	Lactate	0.9	0.035836	-0.09	0.12	-0.75	Lactate*	0.8	0.008790	3.26	0.88	3.71	Lactate	0.8	0.178160	0.05	0.1	0.50429
	Mannose-6-phosphate	0.6	0.249949	-0.03	0.80	-0.04	Mannose-6-phosphate	1.0	0.007688	1.65	0.86	1.92	Mannose-6-phosphate	0.9	0.111905	0.02	0.14	0.1315286
	Oxoadipic acid	0.5	0.974596	0.43	0.44	0.98	Oxoadipic acid*	1.0	0.006120	1.61	0.67	2.40	Oxoadipic acid	0.9	0.088934	-0.78	0.55	-1.4216
	Proline	0.6	0.569723	0.08	0.10	0.81	Proline	0.9	0.039952	2.02	0.99	2.04	Proline	0.7	0.464646	0.34	0.26	1.3256154

Biomarker analysis comparing WT vs. Mut (GHD), Mutant (GHD) vs. Mutant (GH), and WT vs. WT (GH). Metabolites are categorized as GHD Biomarkers, GH treatment-responsive biomarkers, and GH treatment-specific responsive Biomarkers. The table includes AUC, *p*-value, and Log_2_FC.

The GHD biomarkers identified in the study included significant metabolomic alterations across different metabolites. These biomarkers exhibited notable log2 fold changes (Log2 FC), such as 3-hydroxybutyric acid (2.37), AMP (1.87), fumarate (2.44), glucose (-2.16), glutamate (1.70), glycine (1.80), guanidinosuccinic acid (1.85), guanine (2.04), hydroxyproline (-1.85), hypoxanthine (2.65), inosine (3.66), kynurenine (-2.84), leucine (1.83), methionine (3.76), methionine sulfoxide (2.35), pyridoxamine (2.29), pyruvate (1.96), tryptophan (1.52), tyrosine (4.46), xanthine (-1.67), and xylose-5-phosphate (-3.37) (**Table 2**).

The response to GH treatment was characterized by significant changes in several key metabolites. Notable among these were α-ketoglutarate (Log2 FC: 1.86), fructose-6-phosphate (Log2 FC: 2.71), glucose-6-phosphate (Log2 FC: 1.72), lactate (Log2 FC: 3.26), mannose-6-phosphate (Log2 FC: 1.65), oxoadipic acid (Log2 FC: 1.61), and proline (Log2 FC: 2.02).

### Significant metabolic pathway associated with GH treatment responsive biomarkers alterations in Pit-1^^K216E^ mice

**Figure 7:**
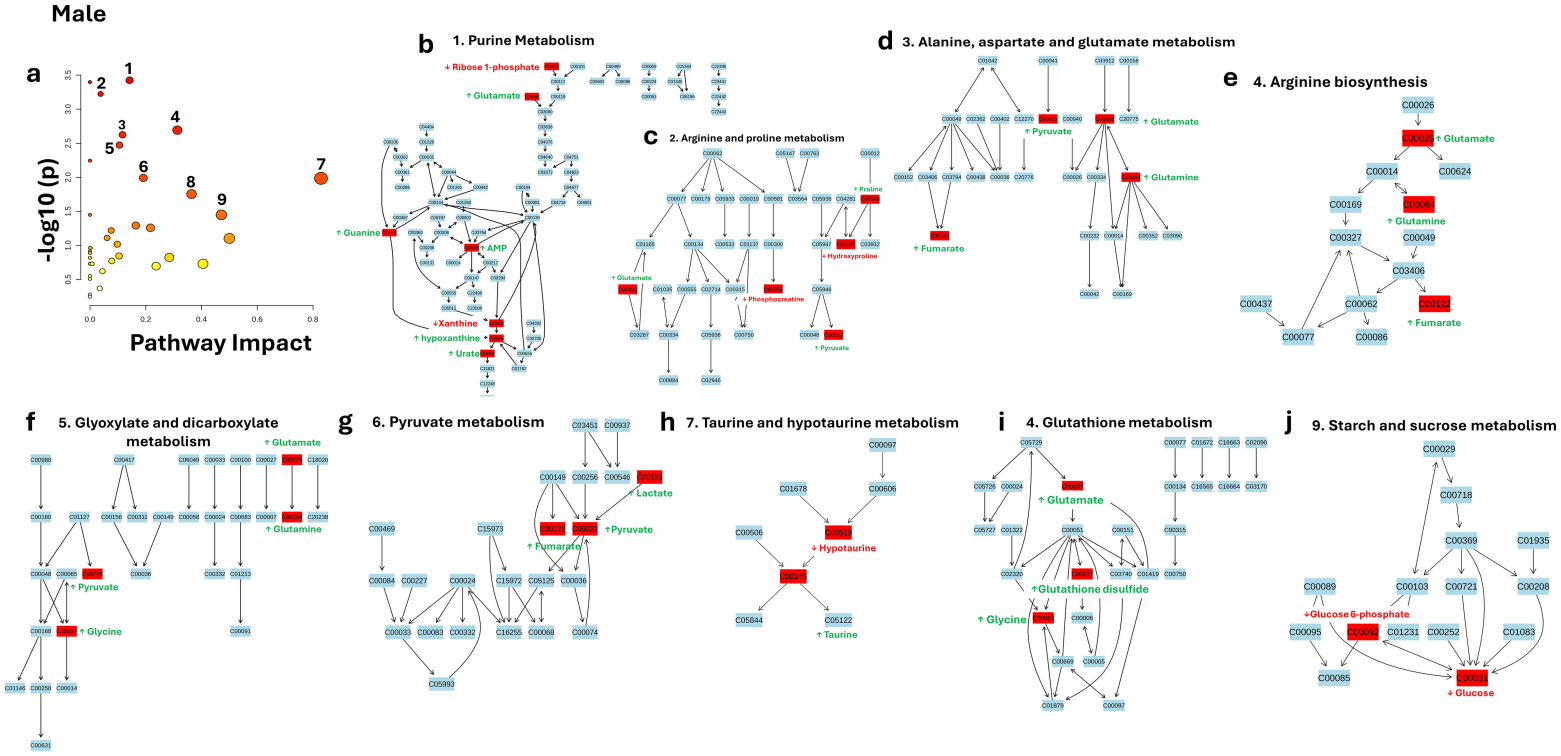
Pathway Analysis of GH Treatment Responsive Biomarkers in Male Mice. This figure presents a pathway analysis of GH Treatment Responsive Biomarkers in males using KEGG pathways. **(a)** Bubble plot showing metabolic pathways ranked by significance (-log10(p)) and pathway impact scores. **(b–j)** Detailed pathway maps highlight key metabolic pathways, with green indicating upregulation and red indicating downregulation of metabolites. Analyzed pathways include: (1) Purine Metabolism, (2) Arginine and Proline Metabolism, (3) Alanine, Aspartate, and Glutamate Metabolism, (4) Arginine Biosynthesis, (5) Glyoxylate and Dicarboxylate Metabolism, (6) Pyruvate Metabolism, (7) Taurine and Hypotaurine Metabolism, (8) Glutathione Metabolism, and (9) Starch and Sucrose Metabolism.

In this study, GH Treatment Responsive Biomarkers metabolites were used for pathway analysis to explore the impact of GHD on metabolic pathways. GH Treatment Responsive Biomarkers are defined as metabolite levels that were different in GHD and WT mice and Mut mice treated with GH. The metabolite levels in WT mice serve as the baseline, with any deviations in the GHD Mut mice representing metabolic disruptions induced by the GHD condition. The pathway analysis using KEGG on GH Treatment Responsive Biomarkers in male mice revealed several significantly impacted metabolic pathways, as illustrated in **Figure 7** and detailed in **Table 3**.

**Table 3:** Pathway Enrichment Analysis of GH Treatment Responsive Biomarkers in Male Mice.

Male									
	Pathway	Total	Expected	Hits	Raw p	Log_10_ (*p*)	Holm adjust	FDR	Impact
1	Purine metabolism	71	1.441	7	0.000375	3.426	0.030	0.016	0.142
2	Arginine and proline metabolism	36	0.731	5	0.000597	3.224	0.047	0.016	0.038
3	Alanine, aspartate and glutamate metabolism	28	0.568	4	0.002027	2.693	0.156	0.038	0.313
4	Arginine biosynthesis	14	0.284	3	0.002372	2.625	0.180	0.038	0.117
5	Glyoxylate and dicarboxylate metabolism	32	0.650	4	0.003363	2.473	0.252	0.045	0.106
6	Pyruvate metabolism	23	0.467	3	0.010196	1.992	0.744	0.092	0.191
7	Taurine and hypotaurine metabolism	8	0.162	2	0.010354	1.985	0.745	0.092	0.829
8	Glutathione metabolism	28	0.568	3	0.017609	1.754	1.000	0.141	0.364
9	Starch and sucrose metabolism	15	0.305	2	0.035542	1.449	1.000	0.237	0.471

Pathway enrichment analysis of GH treatment responsive biomarkers in male mice, listing pathways impacted by GH treatment. Columns include the total number of metabolites per pathway, expected hits, observed hits, raw *p*-values, log_10_(p), Holm-adjusted p-values, false discovery rate (FDR), and impact scores. Pathways are ranked based on statistical significance and biological impact.

The bubble plot in (**Figure 7a**) highlights the pathways based on their statistical significance and impact scores. Purine Metabolism emerged as the most significant pathway, with a p-value of 0.000375 (-log10(p) = 3.426) and an impact score of 0.142 (**Figure 7b**). This pathway is characterized by several metabolites that were found to be higher in WT compared to Mut. Specifically, glutamate (log2 FC = 6.4), guanine (log2 FC = 2.63), aMP (log2 FC = 3.15), hypoxanthine (log2 FC = 10.71), and urate (log2 FC = 4.08) were all elevated in WT mice. Conversely, ribose 1-phosphate (log2 FC = -3.15) and xanthine (log2 FC = - 4.60) were lower in WT compared to Mut mice. Arginine and proline metabolism (**Figure 7c**) was the second most significant pathway, with a p-value of 0.000597 (-log10(p) = 3.224) and an impact score of 0.0384. This pathway shows significant metabolic alterations, with proline (log2 FC = 13.04), glutamate (log2 FC = 6.40), and pyruvate (log2 FC = 4.11) being higher in WT compared to Mut. Conversely, hydroxyproline (log2 FC = -3.12) and phosphocreatine (log2 FC = -7.22) were lower in WT compared to Mut, reflecting the complex metabolic shifts within this pathway.

Alanine, aspartate, and glutamate metabolism (**Figure 7d**) ranked third in significance, with a p-value of 0.00203 and an impact score of 0.313. The impact induced by the GHD condition led to significant changes in metabolite levels within this pathway. Specifically, in WT mice, the metabolites pyruvate (log2 FC = 4.11), fumarate (log2 FC = 5.94), glutamate (log2 FC = 6.40), and glutamine (log2 FC = 3.76) were all higher compared to Mut mice. Arginine biosynthesis (**Figure 7e**) was another significantly impacted pathway, with a p-value of 0.00237 and an impact score of 0.117. The impact induced by the GHD condition led to notable changes in metabolite levels within this pathway. Specifically, in WT mice, the metabolites glutamate (log2(FC) = 6.40), glutamine (log2(FC) = 3.76), and fumarate (log2(FC) = 5.94) were all higher compared to Mut. Glyoxylate and dicarboxylate metabolism (**Figure 7f**) were significantly impacted, with a p-value of 0.0033628 and an impact score of 0.10582. The impact induced by the GHD condition led to notable changes in metabolite levels within this pathway. In WT mice, the metabolites glutamate (log2(FC) = 6.40), glutamine (log2(FC) = 3.76), pyruvate (log2(FC) = 4.11), and glycine (log2(FC) = 2.30) were all higher compared to Mut. Pyruvate metabolism (**Figure 7g**) was another significantly impacted pathway, with a p-value of 0.010196 and an impact score of 0.19137. The impact induced by the GHD condition caused significant changes in metabolite levels within this pathway. Specifically, in WT mice, the metabolites lactate (log2(FC) = 12.33), pyruvate (log2(FC) = 4.11), and fumarate (log2(FC) = 5.94) were all higher compared to Mut. Taurine and hypotaurine metabolism (**Figure 7h**) were significantly impacted, with a p-value of 0.010354 and an impact score of 0.82857, the highest among the pathways analyzed. The impact induced by the GHD condition resulted in notable changes in metabolite levels within this pathway. In WT mice, taurine (log2(FC) = 4.20) was higher compared to Mut, while hypotaurine (log2(FC) = -2.79) was lower compared to Mut, reflecting altered metabolic states in the GHD Mut mice. Glutathione metabolism (**Figure 7i**) was significantly impacted, with a p-value of 0.017609 and an impact score of 0.36435. The impact induced by the GHD condition led to substantial changes in metabolite levels within this pathway. Specifically, in WT mice, glutamate (log2(FC) = 6.40) and glycine (log2(FC) = 2.30) were higher compared to Mut, while glutathione disulfide (log2(FC) = -3.91) was lower compared to Mut, suggesting disruptions in this pathway in the GHD Mut mice.

**Figure 8:**
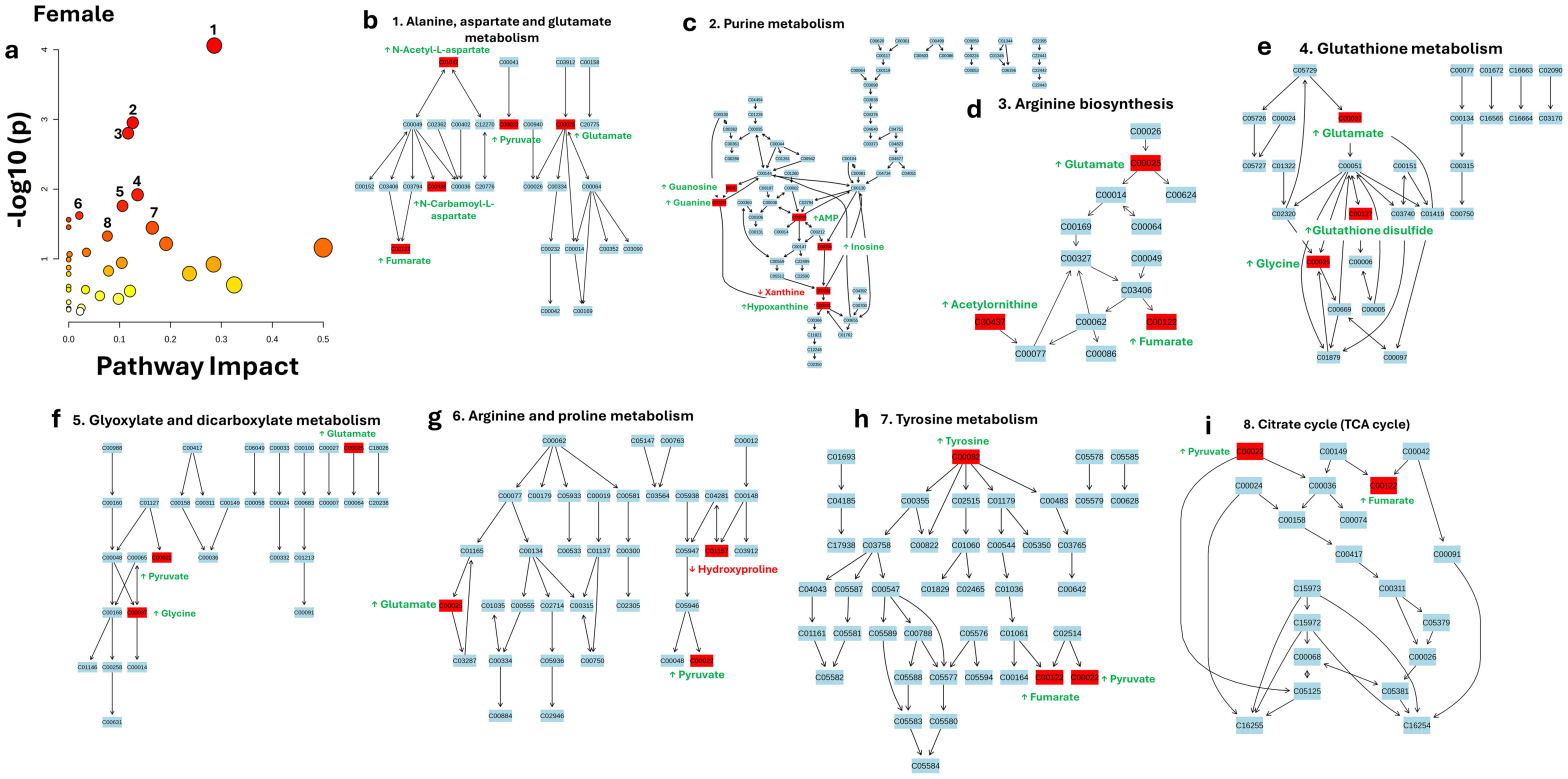
Pathway Analysis of GH Treatment Responsive Biomarkers in Female Mice. This figure presents a pathway analysis of GH Treatment Responsive Biomarkers in females using KEGG. **(a)** Bubble plot showing metabolic pathways ranked by significance (-log10(p)) and pathway impact scores. **(b–i)** Pathway maps highlighting key metabolic pathways, with green indicating upregulation and red indicating downregulation of metabolites. Analyzed pathways include: (1) Alanine, Aspartate, and Glutamate Metabolism, (2) Purine Metabolism, (3) Arginine Biosynthesis, (4) Glutathione Metabolism, (5) Glyoxylate and Dicarboxylate Metabolism, (6) Arginine and Proline Metabolism, (7) Tyrosine Metabolism, and (8) Citrate Cycle (TCA Cycle).

For females, several pathways were also significantly affected (**Figure 8a**). Alanine, aspartate, and glutamate metabolism (**Figure 8b**) was the most significantly impacted pathway, with a p-value of 8.73E-05 ([-log_10_(p) = 4.059]) and an impact score of 0.28606, as detailed in **Table 4**. Key metabolites in this pathway were higher in WT compared to the GHD Mut mice, including N-Acetylaspartate (FC = 1.75), pyruvate (FC = 1.96), N-carbamoyl-L-aspartate (FC = 1.75), glutamate (FC = 1.70), and fumarate (FC = 2.44). Purine metabolism (**Figure 8c**) was the second most significantly impacted pathway, with a p-value of 0.0011129 ([-log_10_(p) = 2.9535]) and an impact score of 0.12595. In this pathway, several metabolites were higher in WT compared to the GHD Mut mice, including guanosine (FC = 1.70), guanine (FC = 2.04), AMP (FC = 1.87), inosine (FC = 3.66), and hypoxanthine (FC = 2.65). Conversely, xanthine (FC = -1.67) was lower in WT compared to the Muts, indicating elevated levels in the GHD Mut mice. Arginine biosynthesis (**Figure 8d**) was the third most significantly impacted pathway, with a p-value of 0.0015777 ([-log_10_(p) = 2.802]) and an impact score of 0.11675. In this pathway, key metabolites such as glutamate (FC = 1.70), acetylornithine (FC = 2.31), and fumarate (FC = 2.44) were higher in WT compared to the GHD Mut mice. Glutathione metabolism (**Figure 8e**) was the fourth most significantly impacted pathway, with a p-value of 0.012035 ([-log_10_(p) = 1.9196]) and an impact score of 0.13537. In this pathway, metabolites including glutamate (FC = 1.70), glutathione disulfide (FC = 2.76), and glycine (FC = 1.80) were higher in WT compared to the GHD Mut mice. Glyoxylate and dicarboxylate metabolism (**Figure 8f**) was the fifth most significantly impacted pathway, with a p-value of 0.017382 ([-log_10_(p) = 1.7599]) and an impact score of 0.10582. Metabolites such as glutamate (FC = 1.70), pyruvate (FC = 1.96), and glycine (FC = 1.80) were higher in WT compared to the GHD Mut mice. Arginine and proline metabolism (**Figure 8g**) was the sixth most significantly impacted pathway, with a p-value of 0.023872 ([-log_10_(p) = 1.6221]) and an impact score of 0.02093. In this pathway, pyruvate (FC = 1.96) and glutamate (FC = 1.70) were higher in WT compared to the GHD Mut mice, while hydroxyproline (FC = -1.85) was lower in WT compared to the Muts. Tyrosine metabolism (**Figure 8h**) was the seventh most significantly impacted pathway, with a p-value of 0.035772 ([-log_10_(p) = 1.4465]) and an impact score of 0.16435. Key metabolites in this pathway, including tyrosine (FC = 4.46), fumarate (FC = 2.44), and pyruvate (FC = 1.96), were all higher in WT compared to the GHD Mut mice, indicating reduced levels in the Muts. The citrate cycle (TCA cycle) (**Figure 8i**) was the eighth most significantly impacted pathway, with a p-value of 0.04704 ([-log_10_(p) = 1.3275]) and an impact score of 0.07615. Within this pathway, pyruvate (FC = 1.96) and fumarate (FC = 2.44) were found to be higher in WT compared to the GHD Mut mice.

**Table 4:** Pathway Enrichment Analysis of GH Treatment Responsive Biomarkers in Female Mice.

Female									
	Pathway	Total	Expected	Hits	Raw p	Log10 (p)	Holm adjust	FDR	Impact
1	Alanine, aspartate and glutamate metabolism	28	0.495	5	0.00009	4.0590	0.007	0.007	0.286
2	Purine metabolism	71	1.255	6	0.00111	2.9535	0.088	0.042	0.126
3	Arginine biosynthesis	14	0.248	3	0.00158	2.8020	0.123	0.042	0.117
4	Glutathione metabolism	28	0.495	3	0.01204	1.9196	0.927	0.241	0.135
5	Glyoxylate and dicarboxylate metabolism	32	0.566	3	0.01738	1.7599	1.000	0.278	0.106
6	Arginine and proline metabolism	36	0.637	3	0.02387	1.6221	1.000	0.314	0.021
7	Tyrosine metabolism	42	0.743	3	0.03577	1.4465	1.000	0.318	0.164
8	Citrate cycle (TCA cycle)	20	0.354	2	0.04704	1.3275	1.000	0.376	0.076

Pathway enrichment analysis of GH treatment responsive biomarkers in female mice, listing pathways impacted by GH treatment. Columns include the total number of metabolites per pathway, expected hits, observed hits, raw *p*-values, log_10_(p), Holm-adjusted p-values, false discovery rate (FDR), and impact scores. Pathways are ranked based on statistical significance and biological impact.

## Discussion

Human growth is dependent on GH, a 191-amino-acid polypeptide hormone synthesized by the anterior lobe of the pituitary gland. Insufficient production of GH results in GHD, which manifests as impaired growth and SS in childhood (27). Diagnosing GHD requires a complex evaluation that includes laboratory testing and radiologic imaging. GH release results in peripheral synthesis and secretion of IGF-I, a marker of GH action with relatively high specificity but low sensitivity (28). The diagnostic tests for GHD are laborious and invasive, and none have the reproducibility and precision required to diagnose either a sufficient or insufficient GH state (12, 18, 20, 29, 30). Further, the lack of sensitivity of current testing results in a failure to capture subtle metabolic changes, especially in adults, resulting in delayed therapeutic interventions and an increased risk of long-term complications (3, 4). Although GH is critical for childhood growth, it is crucial in adults to regulate lipid metabolism, body composition, bone mass, and possibly cognition (31). Hence, monitoring the efficacy of GH therapy in children focuses on linear growth and monitoring adults, including weight, serum IGF-I levels, lipid profile, and Hemoglobin A1c (HbA1c). Although these parameters are used clinically, they often lead to inconclusive benefits (30). In children, serum IGF-I measurements are used as a ‘safety’ marker for overtreatment, which has been linked to pathologies associated with the GH excess of gigantism and the potential for future malignancies. However, whether serum IGF-I is a ‘safety’ marker is controversial (32). Treatment of adult GHD decreases cardiovascular risk factors but can lead to insulin resistance and a metabolically unhealthy phenotype, and the balance between overtreatment or undertreatment of adults with GHD and cardiovascular risk factors remains unknown (33). The use of metabolomics in GHD is a potentially superior approach to identifying states of sufficiency and insufficiency (18, 20, 22).

This study sought to explore how metabolomics profiling can be used in diagnosing GHD. We utilized a mouse model of complete GH insufficiency that allows auxological measures of treatment success to be correlated with clinically relevant metabolomic biomarkers. We used a novel mouse model carrying a point mutation in the *Pit-1* gene, which recapitulates a mutation previously identified in humans, resulting in GHD (24).

The Pit-1^^K216E^ mouse model exhibits phenotypic features of GHD, including small size, abnormal body composition, and low serum GH. The phenotypic, hormonal, and metabolic features mirror the clinical phenotype observed in humans with *PIT-1* mutations and align with the well-established role of the *PIT-1* gene in the development and function of pituitary somatotrophs. These characteristics make the Pit-1^^K216E^ mouse model a valuable tool for exploring the underlying hormonal and metabolic changes associated with the deficiency and potential treatments (34).

In humans, the Pit-1^^K216E^ mutation is associated with deficiencies in GH, PRL, and TSH. GHD is consistently present, while TSH deficiency may co-occur with GHD or develop over time. PRL deficiency is also observed; however, it is not routinely assessed in clinical settings for hypopituitarism, as its clinical impact is typically limited to lactation failure and does not contribute to broader manifestations.

A study investigating patients with multiple pituitary hormone deficiencies identified an independent effect of PRL on IGF-1 status through multiple regression analysis, though no direct correlation was found between actual PRL levels and IGF-1 levels in panhypopituitarism (35). Earlier research demonstrated the effects of infused PRL on IGF-1 synthesis in the liver and explored PRL’s role in cell signaling, further supporting the idea of mechanistic interactions between PRL and IGF-1 regulation. However, the clinical significance of these findings appears limited in the context of panhypopituitarism (36, 37).

In our study, the linear growth and metabolic phenotypes of the Pit-1^^K216E^ mice were effectively normalized following recombinant GH (rGH) treatment, mirroring the therapeutic outcomes observed in humans with this mutation. This underscores the efficacy of rGH treatment in addressing the primary deficits caused by GHD in this model. Although we cannot entirely exclude the possibility of PRL deficiency contributing to the phenotype, its role appears to be minor and unlikely to have a clinically significant impact on growth and metabolism, as observed in humans.

Patients with the Pit-1^^K216E^ mutation exhibit a range of thyroid function, from TSH sufficiency to insufficiency over time. This mutation, along with others in the POU1F1 gene, is associated with low-normal serum T4 levels and a blunted TSH response to TRH stimulation, as noted by Cohen et al. Thyroid function in children with hypopituitarism may initially appear normal but can deteriorate over time, supporting the recommendation for ongoing screening for additional pituitary hormone deficiencies in such patients (24).

In our study, the Pit-1^^K216E^ mouse model exhibited elevated TSH levels alongside normal serum T3 and T4 levels. This discrepancy could reflect the spectrum of TSH levels associated with the mutation, with elevated TSH levels not yet observed in the limited human cases described. Alternatively, the elevated TSH could arise from Pit-1-independent thyrotrophs persisting in the rostral tip of the pituitary gland. Despite these findings, the model remains a valuable tool for studying GHD with normal thyroid function. Further studies are required to elucidate how this mutation influences thyroid function without immediately altering thyroid hormone levels, as also noted by Pfäffle et al. and others (38–40).

Mutations in the Pit-1 gene do not affect gonadotroph function, which is consistent with prior observations and explains why gonadotropin levels were not measured in this study. Patients with these mutations often exhibit normal or delayed pubertal development. In our study, mutant mice displayed small but otherwise normal external genitalia. The breeding challenges encountered were not due to gonadotroph dysfunction but rather the small size of the mice, which necessitated the use of HydroGel^®^ for nourishment to ensure survival.

The observed sexual dimorphism in the metabolomic profiles likely reflects the inherent physiological differences in GH regulation and its downstream effects. GH exerts sexually dimorphic effects on metabolism, differentially impacting lipid, amino acid, and carbohydrate metabolism pathways. These differences may be shaped by the distinct patterns of GH secretion and receptor sensitivity in males and females. For instance, males typically exhibit pulsatile GH secretion, while females have more continuous secretion, influencing metabolic pathways in sex-specific ways.

The five common GH-deficient mouse models including GH^-/-^, GHRH^-/-^, lit/lit, Ames, and Snell have been developed to study phenotypic abnormalities related to GHD (5). These models exhibit deficiencies in GH and IGF-I, accompanied by hallmark features including reduced growth, increased insulin sensitivity, and increased adiposity (5, 41). The Pit-1^^K216E^ mouse model expands the group of GHD models, offering unique insights into the GHD associated with a *Pit-1* gene mutation. This model demonstrates auxological and biochemical similarities to human GHD caused by POU1F1 mutations, such as SS, low IGF-I levels, and increased adiposity, and responds to GH treatment with an increase in body weight, length, serum IGF-I levels, and body composition (e.g., increased lean mass relative to fat mass). By comparing the metabolic features of the Pit-1^^K216E^ mouse model with other established GHD models, it becomes evident that the metabolic changes observed, such as decreased adiposity, align with those reported in different models of GHD, supporting the attribution of these changes specifically to GHD Moreover, the responsiveness of the Pit-1^^K216E^ mouse to GH treatment further validates its utility as a translational tool for understanding metabolic dysregulation in GHD and assessing therapeutic interventions.

Metabolomic studies have emerged as powerful tools for identifying biomarkers that offer valuable insights into the biochemical changes associated with various conditions, including GHD (17). This study aimed to explore the metabolomic alterations resulting from GHD and the subsequent response to GH treatment using the Pit-1^^K216E^ mouse model. This model offers a unique opportunity to investigate the metabolomic profile of GHD in a controlled setting, enabling a systematic analysis of metabolic differences across multiple conditions.

By comparing the metabolomic profile of WT mice (GH-sufficient condition) with Mut mice (GHD condition), we identified a unique set of biomarkers associated with GHD. These GHD biomarkers represent metabolites that exhibit significant changes based on stringent selection criteria, including control for GH treatment of WT mice, ensuring that only the most robust and biologically relevant alterations were considered. These metabolic changes provide critical insights into the fundamental disruptions caused by GHD and serve as potential biomarkers of GHD while highlighting key pathways the disorder affects.

Despite sex-specific differences, several metabolites were common between male and female mice, including 3-hydroxybutyric acid, fumarate, AMP, glutamate, and methionine, which are essential to energy and amino acid pathways. These common biomarkers indicate that GH deficiency leads to core metabolic disruptions affecting both sexes, particularly in energy metabolism. The consistent alteration of metabolites such as glucose and pyruvate further highlights a fundamental disruption in glucose utilization and glycolytic pathways, aligning with the well-established impact of GH deficiency on growth and energy balance (42, 43).

In males, the identified GHD biomarkers revealed major disruptions across metabolic activities and reflected the broad physiological impact of GHD. These metabolites point to significant alterations in energy production, nucleotide synthesis, amino acid metabolism, and oxidative stress regulation, all critical for normal growth and development. Key energy-related metabolites, including creatine phosphate, glucose, glucose-6-phosphate, and lactate, suggest impaired cellular energy production and storage, consistent with the reduced energy efficiency seen in GHD (42, 44). Disruptions in the TCA cycle and glycolysis are further indicated by the presence of 3-hydroxybutyric acid, AMP, fumarate, and pyruvate, emphasizing altered energy metabolism pathways. Nucleotide metabolism is also significantly impacted, as shown by including cytidine, cytosine, purine, ribose phosphate, guanine, hypoxanthine, and xanthine. These metabolites are crucial for DNA and RNA synthesis, essential cell growth, and tissue repair processes, and they are often compromised in individuals with GHD. Amino acid metabolism is also significantly disrupted. Metabolites such as glutamine, glutamate, glycine, leucine, methionine, tyrosine, proline, tryptophan, kynurenine, and hydroxyproline are involved in protein synthesis, neurotransmission, and tissue repair. Their dysregulation may contribute to decreased lean muscle mass and altered body composition observed in GHD (8). In addition, markers like glutathione, methionine sulfoxide, and uric acid suggest increased oxidative stress and altered redox balance (45). These changes may exacerbate cellular damage and further impair growth and tissue function in GHD. The known significance of these pathways to growth and metabolic function associated with GHD gives credence to their use as biomarkers of GHD.

In females, our study identified key GHD biomarkers in mice, offering new insights into distinct sex-specific metabolic alterations associated with GHD. These metabolites reflect core disruptions in pathways critical for energy metabolism, amino acid processing, and cellular maintenance. The presence of unique biomarkers such as glucosamine, homocitrulline, N-acetylaspartate, N-acetyl-L-ornithine, and phenylpropanolamine in the female GHD profile suggests distinct metabolite changes not observed in males. For example, glucosamine is involved in glycosaminoglycan biosynthesis, crucial in joint health and cartilage formation (46, 47). Its alteration may signal early metabolic disturbances in connective tissue, potentially linking GH deficiency to skeletal or joint complications, specifically in adult females. Further, the presence of metabolites related to purine metabolism and the urea cycle in females could reflect a heightened sensitivity to disruptions in nitrogen metabolism, which may contribute to differences in how females experience and adapt to GH deficiency over time. The observed sex differences may be driven by differences in hormone regulation between males and females, as estrogen levels are known to modulate energy metabolism and may interact differentially in the GH axis.

Assessing the efficacy of GH treatment in GHD is challenging due to the difficulty in identifying biomarkers that capture both GHD-related changes and the response to GH therapy. Our mouse model provides a unique platform to identify biomarkers specific to GHD while simultaneously evaluating their impact on GH treatment. These biomarkers denote GH sufficiency and can be referred to as ‘GH Treatment Responsive Biomarkers’, as they specifically respond to GH treatment in the context of GHD, are present in (WT), and do not change in the WT group treated with GH. For example, hydroxybutyric acid (3-HB), a ketone body produced during fat metabolism (48), decreased in GHD, suggesting a reduced reliance on fatty acid oxidation for energy in a GHD state. With GH treatment, 3-HB levels increased, indicating a restoration of fat metabolism and energy homeostasis, likely through enhanced lipolysis.

Similarly, AMP, a crucial energy-sensing molecule (49, 50), was observed to be lower in GHD when compared with the GH-sufficient state. This reduction may reflect a diminished cellular energy demand and altered metabolic priorities, as GHD often leads to reduced anabolic activity and overall energy expenditure (49). However, it is important to note that AMP levels typically rise under conditions of energy stress when ATP is depleted and ADP is converted to AMP by adenylate kinase (49). This contradiction suggests a complex interplay in GHD where reduced anabolic activity might mask traditional energy stress responses. Following GH treatment, AMP levels increased with increased energy demands as GH treatment normalized anabolic processes.

Fumarate, an intermediate of the TCA cycle (51), was low in GHD, implying a potential slowing of the TCA cycle, perhaps reflecting lower energy demands. These observations suggest that GHD leads to significant disruptions in energy metabolism as GH plays a crucial role in rebalancing these processes, promoting the efficient use of fat and glucose as energy sources, which are essential for anabolic growth and overall metabolic health.

In addition to the previously identified biomarker groups, our mouse model enabled us to identify a third category, termed GH Treatment-Specific Responsive Biomarkers. These biomarkers, indicating a sufficiency of GH, increased exclusively in response to GH treatment in the context of GHD while remaining unaffected in GH-deficient conditions and unchanged in the WT group treated with GH. Notably, glutathione disulfide was classified as a GH Treatment-Specific Responsive Biomarker in males, as it was unchanged between WT and GHD conditions, but identified as a GH Treatment Responsive Biomarker in females, indicating potential differences in its role in oxidative stress regulation between sexes that differentially respond to GH treatment. This suggests the presence of sex-specific metabolic responses to GH therapy in the context of GHD.

In males, identifying glutathione disulfide as a GH Treatment Responsive Biomarker indicates that oxidative stress may be more prominently addressed only after GH treatment. This suggests that, in GHD males, oxidative stress is not detected until the metabolic recovery associated with GH therapy. GH thus plays a significant role in re-establishing oxidative balance in males, with glutathione disulfide crucial for neutralizing reactive oxygen species and maintaining redox homeostasis (52). This suggests that GH treatment is essential for activating pathways involved in oxidative stress regulation, where glutathione disulfide acts as a critical component in restoring cellular redox equilibrium (53).

Glutathione disulfide is identified as a GH Treatment Responsive Biomarker in females as well, as it is differentially expressed in the GHD state and significantly changes following GH treatment. This indicates that oxidative stress is detected in GHD and responds to GH therapy in females. The consistent expression of this biomarker across both GHD and GH-treated conditions suggests that females may experience heightened oxidative stress during GHD, which persists until GH treatment restores metabolic equilibrium (54).

This example highlights the importance of recognizing potential sex-specific responses to GHD and treatment, particularly in oxidative stress. Females may rely on oxidative mechanisms in the absence of GH, while oxidative metabolism in males occurs in response to GH treatment. Understanding these distinctions may help tailor GH therapies to account for these sex-specific differences, leading to more effective, personalized, sex-specific treatment strategies for managing GHD.

To investigate the metabolic disruptions caused by GHD and the effects of GH treatment, we conducted pathway analysis using the KEGG database, which integrates individual metabolite changes into broader biological networks. This approach offers a comprehensive view of the metabolic pathways disrupted by GHD and their modulation in response to GH therapy. Purine metabolism, one of the major pathways affected in both males and females, plays a critical role in synthesizing nucleotides such as ATP and GTP, which are vital for energy transfer, DNA/RNA synthesis, and cellular signaling (55, 56). Disruption of this pathway in GHD suggests a compromised nucleotide pool, impairing cellular energy homeostasis and reducing the availability of purine nucleotides for growth and cell proliferation. This likely contributes to growth impairment and reduced cellular activity, which are critical features of GHD.

Alanine, aspartate, and glutamate metabolism, which were significantly disrupted in both sexes, is involved in the production of amino acids that serve as key intermediates in energy metabolism and neurotransmitter synthesis (57). Alanine and glutamate are critical for glucose metabolism, linking amino acid catabolism to gluconeogenesis (58). The disruption of this pathway suggests a dual impact on energy metabolism and neurotransmitter production, potentially leading to both metabolic inefficiency and cognitive impairment. Uncovering the molecular basis of aspartate metabolism argues for the important role of GH in neuronal development during a critical period in childhood.

In males, taurine and hypo-taurine metabolism alterations suggest a diminished capacity to manage oxidative stress and regulate bile salt formation, both critical for cellular homeostasis and lipid metabolism (59). Disruption of this pathway may lead to additional cellular stress and impaired detoxification, contributing to the metabolic burden of GHD. This may be an essential mechanism regulating the often-seen elevations in lipids and metabolic dysfunction-associated steatotic liver disease (MASLD) in adult GHD and the decreased bile acid synthesis and structural abnormalities in the bile acid canaliculi associated with elevated levels of direct and total bilirubin seen in infants with GHD.

Two additional pathways were notably affected in females: tyrosine metabolism and the TCA cycle. Tyrosine metabolism is critical for synthesizing neurotransmitters such as dopamine, (60), which play essential roles in cognitive function and mood regulation (61). The disruption of tyrosine metabolism in GHD may reflect underlying cognitive and mood changes, which are more pronounced in females. Additionally, disruptions in the TCA cycle, a central hub of cellular respiration (62), suggest impaired mitochondrial energy production, leading to further metabolic inefficiencies.

The metabolic alterations identified in our mouse model align with known disruptions in human GHD, underscoring the potential clinical relevance of these biomarkers. Notably, disturbances in amino acid and purine metabolism, including alterations in hypoxanthine, xanthine, and branched-chain amino acids (BCAAs), have been reported in GHD patients (20). These metabolic changes are associated with oxidative stress, impaired nucleotide turnover, and altered protein metabolism, highlighting the translational significance of our findings (63).

Amino acid imbalances, particularly in glutamate, methionine, glycine, and leucine, are frequently observed in GHD patients, contributing to muscle weakness, nitrogen imbalance, and metabolic inefficiencies (63, 64).Importantly, our study observed that biomarkers such as glycine, pyruvate, and leucine normalized following GH therapy, reflecting the well-documented metabolic effects of GH replacement in humans (20). This normalization supports the potential of these biomarkers for monitoring GH treatment response in GHD.

The observed sex-specific metabolic variations in the Pit-1^K^²¹^6E^ mouse model may be partially influenced by hormonal differences, particularly estrogen and testosterone. Estrogen is known to regulate lipid metabolism, glucose homeostasis, and amino acid turnover, contributing to metabolic distinctions between males and females (65). Testosterone, on the other hand, plays a key role in muscle protein synthesis and energy metabolism, which could impact the metabolic profile of male mice (66). These hormonal influences may explain the differential response to GH treatment observed in this study (67). Further investigations measuring circulating hormone levels and their interactions with GH signaling could provide deeper insights into the mechanistic basis of these sex differences.

In conclusion, The Pit-1^^K216E^ mouse model has proven to be an essential tool for studying GHD conditions in a controlled experimental setting. Notably, the Pit-1^^K216E^ model retains a functional GH signaling system, as evidenced by the partial restoration of the GHD phenotype following GH treatment and the elevation of IGF-I levels in the serum. Moreover, the feasibility of this model for studying GHD under different experimental conditions, such as baseline GHD and GH-deficient versus GH-treated states, has facilitated the identification of multiple sets of biomarkers. These biomarkers can potentially be used to improve the diagnostic yield in testing for GHD and inform on GH sufficiency, especially in adults lacking precise markers and those children appearing to be unresponsive to GH therapy.

However, it is important to acknowledge the limitations inherent in using animal models to study human diseases like GHD. While the Pit-1^^K216E^ mouse model closely replicates key aspects of human GHD, differences in physiology, lifespan, and metabolism between mice and humans could influence the generalizability of the findings. Additionally, the hormonal regulatory systems in mice, though similar to humans, may respond differently to therapeutic interventions, potentially limiting the direct applicability of these results to human GHD patients. Moreover, metabolomic profiling techniques are inherently subject to variability in sample processing, instrument sensitivity, and data normalization, which could influence metabolite detection and quantification. While this study focuses on specific metabolic pathways and biomarkers, it’s possible that other important pathways may not have been identified.

The complexity of GHD and the broad systemic effects of GH suggest that further research is needed to explore additional biochemical pathways and validate the identified biomarkers in human studies. Future studies should also address the long-term effects of GH treatment and the potential for age- or sex-related differences in metabolic responses, which could further refine diagnostic and therapeutic approaches.

## Data Availability

The raw data supporting the conclusions of this article will be made available by the authors, without undue reservation.
