# Corrigendum: Biomarkers of GH deficiency identified in untreated and GH-treated Pit-1 mutant mice

**DOI:** 10.3389/fendo.2025.1631601

**Published:** 2025-08-07

**Authors:** Sarmed Al-Samerria, Huiting Xu, M. Elena Diaz-Rubio, Joseph Phelan, Chi Su, Keer Ma, Anna Newen, Kiana Li, Sayaka Yamada, Ariel L. Negron, Fredric Wondisford, Sally Radovick

**Affiliations:** ^1^ Department of Pediatrics, University of Arizona College of Medicine, Phoenix, AZ, United States; ^2^ Department of Medicine, Robert Wood Johnson Medical School, Rutgers, The State University of New Jersey, New Brunswick, NJ, United States; ^3^ Rutgers Cancer Institute, Robert Wood Johnson Medical School, Rutgers, The State University of New Jersey, New Brunswick, NJ, United States; ^4^ Department of Medicine, University of Arizona College of Medicine, Phoenix, AZ, United States

**Keywords:** growth hormone deficiency (GHD), biomarkers, PIT-1 mutation, metabolomics, GH treatment, energy metabolism, sex differences

There was a mistake in **Figure 5** as published. **Figure 5** was incorrect and mistakenly showed the same content as Figure 6, rather than the correct figure. The corrected **Figure 5** and its caption appear below.

**Figure 5:**
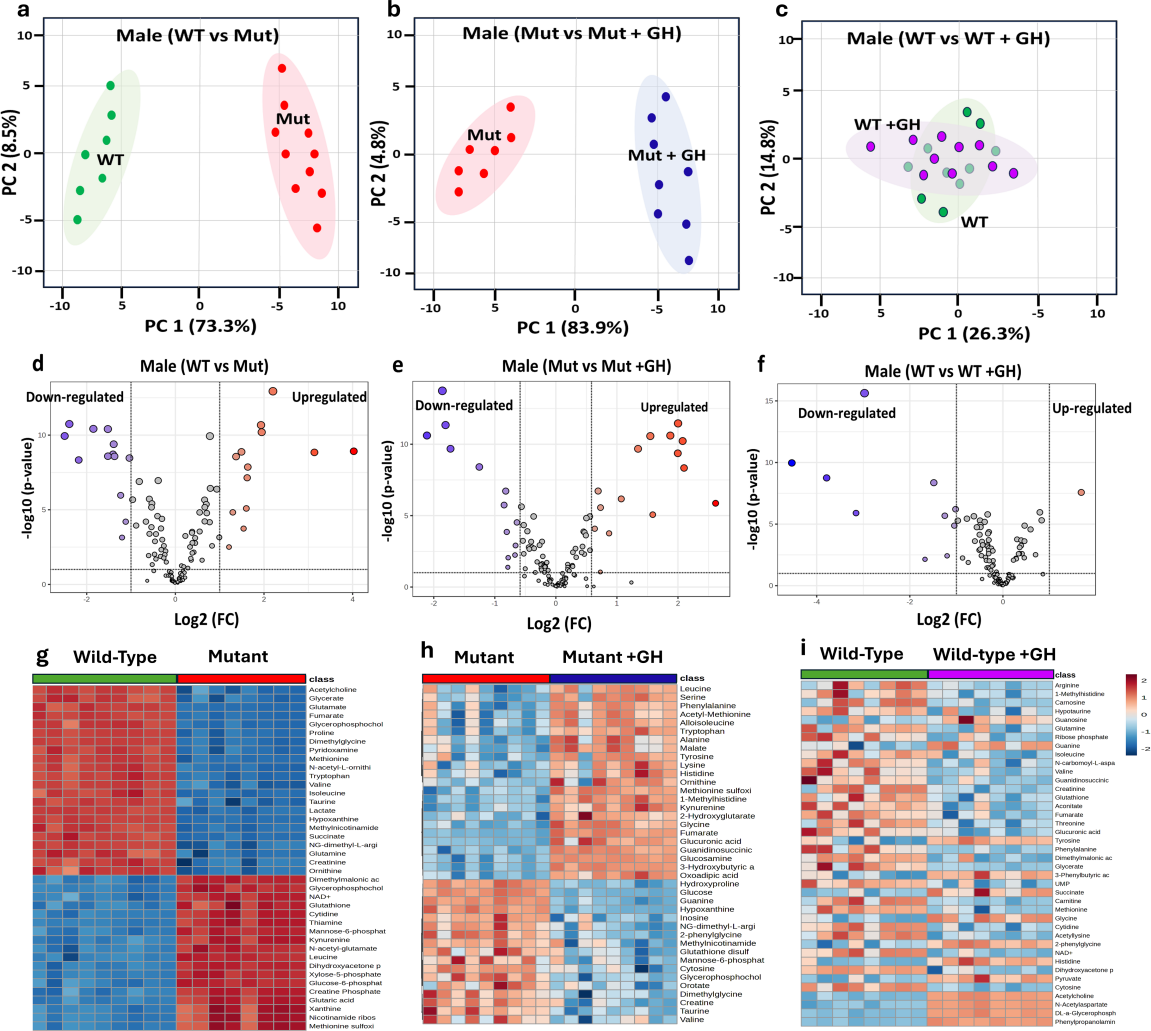
Comparative metabolomic analysis to identify GHD Biomarkers in male mice. This figure examines metabolomic profiles under different GH conditions to identify GHD biomarkers and GH treatment effects. PCA plots **(a–c)** show metabolic distinctions: **(a)** GH sufficient (WT) vs. GHD (Mut), **(b)** GHD (Mut) vs. GH supplemented (Mut + GH), and **(c)** GH sufficient (WT) vs. GH excess (WT + GH). Volcano plots **(d–f)** display differentially expressed metabolites based on Log2 fold change and p-values for the same comparisons. Heatmaps **(g–i)** illustrate key metabolite expression across groups.

The original version of this article has been updated.

